# C_4_ photosynthetic anatomy is associated with higher leaf hydraulic conductance and capacitance in *Alloteropsis semialata*

**DOI:** 10.1111/nph.71221

**Published:** 2026-04-29

**Authors:** Yanmin Zhou, Luke T. Dunning, Hui Liu, Colin P. Osborne

**Affiliations:** ^1^ Plants, Photosynthesis and Soil, School of Biosciences University of Sheffield Sheffield S10 2TN UK; ^2^ Guangdong Provincial Key Laboratory of Applied Botany, Key Laboratory of National Forestry and Grassland Administration on Plant Conservation and Utilization in Southern China South China Botanical Garden, Chinese Academy of Sciences Xingke Road 723 Guangzhou 510650 China; ^3^ Ecology and Evolutionary Biology, School of Biosciences University of Sheffield Sheffield S10 2TN UK

**Keywords:** *Alloteropsis semialata*, C_4_ photosynthesis, climate, evolution, leaf anatomy, leaf hydraulics

## Abstract

- The evolution of C_4_ photosynthesis requires biochemical innovations to be coordinated with anatomical modifications. In many C_4_ species, CO_2_ is concentrated and fixed within leaf bundle sheath tissues, which are enlarged relative to other leaf tissues via cell expansion and the insertion of additional minor veins.
- In this study, we tested the hypothesis that anatomical adaptations for C_4_ photosynthetic carbon assimilation also improve plant–water relations. We measured leaf hydraulics and water retention characteristics in photosynthetically diverse populations of the grass *Alloteropsis semialata*.
- C_4_ individuals of *Alloteropsis semialata* had higher values of leaf capacitance and leaf hydraulic conductance than non‐C_4_ plants. Greater minor vein density contributed to higher values of leaf hydraulic conductance and less negative leaf water potential. Moreover, a greater ratio of bundle sheath area : leaf width and thicker leaves were associated with higher values of leaf capacitance and more negative turgor loss points in C_4_ plants. Further diversification of leaf water relations traits was related to secondary adaptations to climate and polyploid formation.
- These findings are consistent with the hypothesis that anatomical adaptations for C_4_ photosynthesis also improve plant–water relations in grasses and explain how C_4_ photosynthesis expanded the hydrological niche in this species.

## Graphical abstract

Bulk leaf capacitance (*C*_bulk_) in relation to bundle sheath area (BSA) and leaf dimensions in *Alloteropsis semialata* plants differing in photosynthetic type (C3, C3–C4 or C4) and ploidy level (2x, 6x or 12x).

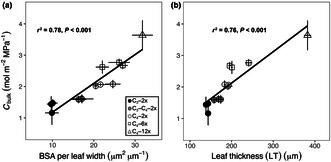

## Introduction

C_4_ photosynthesis has evolved more than 60 times to concentrate CO_2_ around the carboxylating enzyme Rubisco, thereby improving photosynthetic efficiency and maximum carbon fixation rate compared with C_3_ photosynthesis (Sage, 2004; Osborne & Sack, 2012; Liu & Osborne, 2015). The evolution of C_4_ physiology from C_3_ ancestors required changes to the activity and localisation of enzymes and transporters that were coordinated with anatomical specialisation (Osborne & Sack, 2012; Lundgren *et al*., 2019a). The majority of C_4_ plants possess a distinctive anatomical arrangement of cells in the leaf, named Kranz anatomy, in which densely packed veins are wrapped by enlarged bundle sheath tissues containing abundant chloroplasts (Black Jr & Mollenhauer, 1971; Maai *et al*., 2011; Stata *et al*., 2014). Each layer of bundle sheath cells is surrounded by one or two layers of mesophyll cells (Lundgren *et al*., 2014). The unique Kranz anatomy of C_4_ plants enables them to concentrate CO_2_ in the bundle sheath cells around Rubisco while excluding O_2_ to effectively suppress the oxygenation reaction of Rubisco and the energetically costly photorespiratory pathway (Osborne & Sack, 2012; Lundgren *et al*., 2016).

C_4_ photosynthesis offers advantages for carbon fixation under environmental conditions that promote high rates of photorespiration, such as low atmospheric CO_2_, high light and temperature, and aridity or salinity (Edwards *et al*., 2010; Sage *et al*., 2011; Kadereit *et al*., 2012; Christin & Osborne, 2014; Lundgren *et al*., 2016; Zhou *et al*., 2018). In addition, since the C_4_ carbon‐concentrating mechanism (CCM) achieves faster CO_2_ fixation at lower intercellular CO_2_ partial pressures, C_4_ plants typically operate with a lower stomatal conductance (*g*_s_) than C_3_ species. This enables C_4_ grasses to maintain a high net leaf photosynthetic rate (*A*_n_) at lower *g*_s_ and limits water loss, resulting in higher water‐use efficiency (Pearcy & Ehleringer, 1984; Huxman & Monson, 2003; Ghannoum, 2009; Way *et al*., 2014).

Several additional interactions of C_4_ evolution with leaf water relations are less well characterised. First, leaf hydraulic adaptations to hot, dry atmospheres or soil water deficits could lead to anatomical phenotypes favouring the subsequent evolution of C_4_ photosynthesis, that is adaptations to high evaporation rates or drought may be preadaptations for C_4_ photosynthesis (Sage, 2004; Osborne & Sack, 2012; Griffiths *et al*., 2013). Second, the high ratio of bundle sheath tissue to mesophyll tissue (BS : M) required for Kranz anatomy is facilitated by enlarged bundle sheath tissues and greater vein densities in C_4_ plants (Christin *et al*., 2013; Lundgren *et al*., 2014), which could directly influence hydraulic behaviour during the evolutionary transition to C_4_ photosynthesis (Sage, 2004; Osborne & Sack, 2012; Liu & Osborne, 2015). In particular, the increased bundle sheath tissue volumes in C_4_ lineages might contribute to a higher leaf capacitance than in C_3_ species, since these tissues have a role in mediating water fluxes between xylem and mesophyll (Sage, 2001; Osborne & Sack, 2012; Griffiths *et al*., 2013). Additionally, since high vein density is positively related to leaf hydraulic conductance (*K*_leaf_) across non‐C_4_ species, higher vein densities in C_4_ species might cause greater *K*_leaf_ (Osborne & Sack, 2012; Griffiths *et al*., 2013). Finally, the coordinated evolution of plant hydraulics with stomatal conductance could lead to decreases in hydraulic conductance following the evolution of C_4_ photosynthesis (Kocacinar & Sage, 2003, 2004).

Early work in this area showed lower stem hydraulic conductance per unit leaf area (LA) in C_4_ than C_3_ eudicots (Kocacinar & Sage, 2003, 2004). Later modelling indicated that increasing *K*_leaf_ should have little impact on the C_4_ advantage over C_3_ species (Zhou *et al*., 2018). Compared with C_3_ species, C_4_ species have lower *g*_s_, leading to a greater (less negative) leaf water potential (*Ψ*_leaf_). Moreover, this integrated modelling framework predicted that C_4_ species should have an increased (less negative) turgor loss point than closely related C_3_ species and confirmed this empirically in four comparisons (Zhou *et al*., 2018). However, broader surveys focused on grasses showed that a higher ratio of *K*_leaf_ to *g*_s_ is a general feature of C_4_ lineages relative to their C_3_ relatives, leading to less negative *Ψ*_leaf_ and maintenance of photosynthesis during the initial phases of soil drying (Taylor *et al*., 2011; Osborne & Sack, 2012; Baird *et al*., 2025). Across these C_4_ grass species, bundle sheath tissue dimensions are positively related to *K*_leaf_, but vein densities are not (Baird *et al*., 2025). Further empirical work inferred, using comparative analysis, that these hydraulic differences may evolve over time, with young C_4_ lineages having greater leaf capacitance and *K*_leaf_ than their C_3_ relatives, with the advantages of hydraulics diminishing in older C_4_ lineages (Zhou *et al*., 2025).

Ecological factors may also play a role. Besides the effect of photosynthetic type, environmental conditions such as temperature and drought are also important for plant–water relations (Osborne & Sack, 2012; Taylor *et al*., 2014; Liu & Osborne, 2015; Baird *et al*., 2025). Specifically, C_4_ grasses can maintain higher values of *g*_s_, *A*_n_, *Ψ*_leaf_ and *K*_leaf_ during the onset of drought (Taylor *et al*., 2011; Osborne & Sack, 2012; Baird *et al*., 2025) and, across grass species, the ratios of *A*_n_ to *g*_s_, *K*_leaf_ to *g*_s_, and *A*_n_ to *K*_leaf_ scale positively with potential evapotranspiration (Baird *et al*., 2025). Under mild water deficits, mesophyll cell membrane conductance to water is downregulated in C_4_ leaves, thereby enabling *g*_s_ to be maintained and CO_2_ exchange to continue (Márquez *et al*., 2024). As a consequence of these various adaptations, C_4_ grasses tend to occupy drier habitats than related C_3_ species (Osborne & Freckleton, 2009). Therefore, whether the reorganisation of leaf hydraulics associated with C_4_ photosynthesis is directly determined by structure–function relationships or is an indirect consequence of habitat associations remains unclear.

The grass *Alloteropsis semialata* (R. Br.) Hitchc. (Poaceae) shows a high level of recently evolved intraspecific variation in photosynthesis, including intermediate forms (Lundgren *et al*., 2016). Its bundle sheath tissues and vein densities have diversified further as a consequence of recurrent polyploid formation, and its large geographical distribution offers the additional chance to investigate whether plant–water relations adapt to local climatic conditions. This taxon therefore represents an unrivalled model for studying within a single species how the evolution of anatomical features associated with photosynthetic pathways interacts with environmental conditions to influence plant–water relations (Zhou & Osborne, 2024). In this paper, we use *A. semialata* as a model to test the hypotheses that: (i) the evolution of specialised leaf anatomy in this C_4_ plant improves hydraulic function in comparison with the C_3_ type; (ii) larger bundle sheath tissues are associated with greater leaf capacitance, and higher vein densities are linked to greater leaf hydraulic conductance; and (iii) warmer and relatively drier habitats select for plants with an increased leaf capacitance and hydraulic conductance.

## Materials and Methods

### Plant material and growth conditions

Experiments were carried out at the Arthur Willis Environment Centre at the University of Sheffield (Sheffield, UK) between 2021 and 2023. The study included 13 populations of *Alloteropsis semialata* (R. Br.) Hitchc. with various photosynthetic types (C_3_, C_3_–C_4_ intermediate, and C_4_) and ploidy levels (diploid, hexaploid, and dodecaploid) from geographic origins spread across the range of this species (Supporting Information Table S1; Lundgren *et al*., 2015; Bianconi *et al*., 2020). Each population was represented by three replicates. Plants were grown in 1‐l plastic pots containing John Innes No. 2 compost (John Innes Manufacturers Association, Reading, UK) and fertilised once a month with Scotts Evergreen Lawn Food (The Scotts Co., Surrey, UK). All pots were placed into a glasshouse with the following environmental conditions: 60% relative humidity, day : night temperatures, 25°C : 20°C, 500–1500 μmol m^−2^ s^−1^ photosynthetic photon flux density (PPFD) provided by natural light together with four lamps in daytime, and the ambient 400 μmol mol^−1^ CO_2_ concentrations over the span of the experiment.

### Climatic niche determination

The values of 19 bioclimatic variables relating to temperature and precipitation were extracted from the WorldClim database (Fick & Hijmans, 2017; Alenazi *et al*., 2023; Zhou & Osborne, 2024) based on the geographic coordinates of collection locations of populations. Full details were provided in Zhou & Osborne (2024).

### Phylogeny and characterising photosynthetic types

The evolutionary relationships of the samples used in this study were inferred by concatenating 3553 previously generated nuclear marker alignments for 77 *Alloteropsis* accessions (Bianconi *et al*., 2020) and then inferring a phylogeny in RAxML v.8.2.4 (Stamatakis, 2014) with default parameters. This phylogeny was trimmed to include only the accessions relevant to this study, using closely related accessions in the same position of the phylogeny if necessary (proxies were selected based on a larger tree containing 566 *Alloteropsis* individuals inferred using ddRADseq data; Olofsson *et al*., 2021). Finally, the tree was made ultrametric using the chronopl function (*λ* = 1) as part of the ape v.5.2 (Paradis & Schliep, 2019) package in R v.4.2.1 (R Core Team, 2022).

The carbon isotope composition of plant tissues (δ^13^C) is related to the Kranz syndrome (Bender, 1968; Tregunna *et al*., 1970; Smith & Brown, 1973). Thus, it was used to distinguish photosynthetic types, as previously described (von Caemmerer *et al*., 2014; Lundgren *et al*., 2016), such that a sample with δ^13^C higher than −17‰ was considered a C_4_ plant, and those with a value of δ^13^C lower than −17‰ were considered non‐C_4_ plants (Smith & Brown, 1973). To further distinguish plants between C_3_ and C_3_–C_4_ intermediate types, previous physiological and anatomical data were applied (Bianconi *et al*., 2020). The values of δ^13^C (see Table S1) were retrieved from previous studies (Lundgren *et al*., 2016, 2019b; Bianconi *et al*., 2020; Olofsson *et al*., 2021; Alenazi *et al*., 2023). Full descriptions of the δ^13^C estimates were provided in the paper by Alenazi *et al*. (2023).

### Ancestral state reconstruction

Ancestral estimation methods were performed using the R packages ape (Paradis *et al*., 2004), phytools (Revell, 2012), and geiger (Harmon *et al*., 2008). A model of Brownian motion with fastAnc function was used to obtain the maximum likelihood ancestral state reconstructions of leaf anatomical and hydraulic traits (Masters *et al*., 2024). To determine the uncertainty in trait estimations at nodes, the intervals of variance and confidence were calculated (Losos, 1999; Masters *et al*., 2024). Posterior probabilities for ancestral state reconstructions were subsequently mapped onto a phylogeny (Yu *et al*., 2017; Masters *et al*., 2024).

### Leaf anatomy

New fully expanded leaves from each population were sampled between the midrib and the margin for anatomical observations. Leaves were stored in 70% ethanol at 4°C for 2 wk before dehydration and fixation. A single leaf segment was excised from the centre of the leaf blade, which was fixed and dehydrated in ethanol before embedding (Lundgren *et al*., 2019b; Alenazi *et al*., 2023). For all leaf samples, leaf pieces 5–7 mm in length were embedded using methacrylate embedding resin (Technovit 7100; Heraeus Kulzer GmbH, Wehrheim, Germany). After embedding, the transverse sections were cut to 8 μm thickness using a manual rotary microtome (Leica Biosystems, Newcastle, UK) and stained with 1% Toluidine Blue O for 1 min (Sigma‐Aldrich; Lundgren *et al*., 2019b; Alenazi *et al*., 2023). Leaf cross‐sections were imaged using a BX51 fluorescence microscope (Olympus, Hamburg, Germany). The sequences of images were stitched together using the Hugin software (Hugin Development Team, 2015; Alenazi *et al*., 2023). The area of bundle sheath tissue in cross‐section, the number of major and minor veins, and leaf thickness (LT) were measured using the Image‐Pro Plus 6.0 software.

We measured inner bundle sheath area (IBSA) and parenchymatous bundle sheath area (PBSA) in cross‐sections (Fig. S1), and then calculated the total IBSA per leaf width, the total PBSA per leaf width, and mean values of IBSA and PBSA per leaf width (BSA per leaf width). There are several different categories of veins in *A. semialata*, such that the midvein (i.e. the large central vein) is the 1° vein, major veins are 2° veins, and minor veins are the 3°, 4° and 5° veins, respectively (Lundgren *et al*., 2016; Baird *et al*., 2021; Robil *et al*., 2021). Major and minor vein densities were determined as the major and minor vein numbers per unit leaf width, which were assumed to be equivalent to the vein length per LA (Sack & Scoffoni, 2013) in these grasses, which have parallel venation.

### Leaf gas exchange

Leaf gas exchange including light‐saturated photosynthetic rate (*A*_sat_) and light‐saturated stomatal conductance (*g*_sat_) was measured on the youngest fully expanded leaf of well‐watered plants between 8:00 h and 11:30 h on sunny days. To determine photosynthetic light response curves, a portable photosynthesis system based on an open gas exchange system (LI‐6400XT with an LI‐6400‐40 fluorometer head unit; LI‐COR, Lincoln, Nebraska, USA) was used to monitor *A*_n_ as a function of PPFD, which was obtained under the following conditions inside the leaf chamber: leaf temperature of 24 ± 1°C, leaf–air vapour pressure deficit (VPD) of 1.5 ± 0.2 kPa, flow rate of 500 μmol s^−1^ and 400 μmol mol^−1^ CO_2_ reference concentration. The light response curves were obtained with an auto‐program function by setting the values of irradiance to the following sequence: 2000, 1600, 1200, 800, 400, 200, 100, 50, and 0 μmol m^−2^ s^−1^, with a minimum wait time of 120 s and a maximum wait time of 240 s. To ensure the accuracy of gas exchange measurements, the infrared red gas analysers were matched before every light‐level transition, maintaining a reference CO_2_ differential of 50 μmol mol^−1^ (Zhou *et al*., 2020). Collected data were modelled with a nonlinear regression to fit a nonrectangular hyperbola based on Thornley (1998):

(Eqn 1) $$A_{n}=\begin{array}{l} \frac{\phi \text{PPFD}+A_{\mathrm{sat}}-\sqrt{{\left(\phi \text{PPFD}+A_{\mathrm{sat}}\right)}^{2}-4\theta \phi \text{PPFD}A_{\mathrm{sat}}}}{2\theta }\\ -\;R_{d} \end{array}$$

(Eqn 2) $$g_{s}=\begin{array}{l} \frac{\phi_{0}\text{PPFD}+g_{\mathrm{sat}}-\sqrt{{\left(\phi_{0}\text{PPFD}+g_{\mathrm{sat}}\right)}^{2}-4\theta_{0}\phi_{0}\text{PPFD}g_{\mathrm{sat}}}}{2\theta_{0}}\\ +\;g_{0} \end{array}$$

where *A*_n_ is the measured CO_2_ assimilation, *A*_sat_ is the light‐saturated CO_2_ assimilation, *φ* and *φ*_0_ are the apparent quantum yield, *θ* and *θ*_0_ are curvature parameters, which determine the sharpness of the response curve knee, and *R*_d_ is the rate of extrapolated dark respiration. *g*_sat_ was obtained similarly to *A*_sat_ by Eqn 2, where *g*_s_ is the measured stomatal conductance, *g*_sat_ is the light‐saturated stomatal conductance, and *g*_0_ is the initial stomatal conductance (Fig. S2).

### Leaf hydraulic capacity

The bench drying method was used to estimate the parameters of pressure–volume (PV) curves (Fig. S3), including the turgor loss point (*Ψ*_TLP_), the osmotic potential at full turgor (*Ψ*_FT_), the relative water content at turgor loss point (RWC_TLP_), the apoplastic water fraction at full turgor (AWF), the bulk modulus of elasticity (*ε*), and the absolute capacitance per LA, assumed to represent bulk‐leaf capacitance (*C*_bulk_). Mature leaves were cut through the sheath and rehydrated overnight as described by Liu & Osborne (2015). Saturated leaves were cut again with a razor blade the next morning, and LA was measured to determine the absolute capacitance per LA. Both leaf weight and *Ψ*_leaf_ were periodically measured using a 4‐point balance (AE163; Mettler Toledo Ltd, Leicester, UK) and a portable pressure chamber (PMS 1000, PMS Instrument Co., USA), separately. Leaves were initially kept in plastic bags that allowed them to dry slowly until an equilibrium mass, and then allowed to dry on the bench for 10–15 min between measurements. This approach enabled *Ψ*_leaf_ intervals of 0.2–0.3 MPa to be captured until *Ψ*_leaf_ of −3.0 MPa was achieved. Leaf materials were dried in a forced air oven at 60°C at the end of measurements to obtain the leaf dry weight (DW), and relative water content (RWC) was calculated as (FW − DW)/(SW − DW) (Liu & Osborne, 2015). FW is fresh weight, and *SW* is saturated weight. *ε* (MPa) was calculated based on the slope of the initial turgor loss line by Sanders & Arndt (2012):

(Eqn 3) $$\varepsilon =\frac{\Delta \Psi_{\text{leaf}}}{\Delta \mathrm{RWC}}\cdot \mathrm{RWC}$$

where RWC is relative water content (%), and *Ψ*_leaf_ is leaf water potential (MPa). *C*_bulk_ (mol m^−2^ MPa^−1^) was calculated according to the initial slope of the PV relationship (∆RWC/∆*Ψ*_leaf_) that was described by Blackman & Brodribb (2011):

(Eqn 4) $$C_{\text{bulk}}=\frac{\Delta \mathrm{RWC}}{\Delta \Psi_{\text{leaf}}}\cdot \frac{\mathrm{DW}}{\mathrm{LA}}\cdot \frac{\mathrm{SW}}{\mathrm{DW}}\cdot \frac{1}{M}$$

where LA is leaf area (m^2^), and *M* is the molar mass of water (g mol^−1^).

Leaf hydraulic conductance (*K*_leaf_, mmol m^−2^ s^−1^ MPa^−1^) was measured following the evaporative flux method (Brodribb & Holbrook, 2003; Simonin *et al*., 2015) as follows:

(Eqn 5) $$K_{\text{leaf}}=\frac{E}{\Psi_{\text{stem}}-\Psi_{\text{leaf}}}$$

where *E* is the transpiration rate (mmol m^−2^ s^−1^) of leaves, and *Ψ*_stem_ is the stem water potential. Measurements were conducted in the glasshouse growth environment between 8:30 h and 11:00 h on sunny days. Two adjacent leaves were selected, one of which was sampled to measure *E* and *Ψ*_leaf_, and the adjacent leaf was used to determine *Ψ*_stem_. To obtain equilibration between *Ψ*_stem_ and *Ψ*_leaf_, plastic film and aluminium foil were applied to cover one of the leaves overnight, which was used as an assay for *Ψ*_stem_ before subsequent measurements. *E* was measured with the LI‐600 porometer (LI‐600, LI‐COR; Lincoln, Nebraska, USA) because plants were exposed to wind conditions (wind speed was over 2 m s^−1^ at the leaf), near the fan in the glasshouse, enabling us to assume leaf boundary layer conductance (*g*_b_) was greater than *g*_s_ (Brodribb & Holbrook, 2003; Simonin *et al*., 2015). In addition, leaf temperature and orientation, and ambient humidity were recorded during the period of measurements of *E*. Thus, the measured value of *E* by the LI‐600 porometer was as representative as possible of the actual *E* (Brodribb & Holbrook, 2003; Simonin *et al*., 2015). *Ψ*_stem_ and *Ψ*_leaf_ were measured using a portable pressure chamber following Simonin *et al*. (2015). Balancing pressure was determined at the point when xylem sap reached the cut surface of the stem, as verified under a dissecting microscope at ×25 magnification (Simonin *et al*., 2015).

### Statistical analysis

All statistical analyses and figures were done in R v.4.2.1 (R Core Team, 2022). A nonparametric Kruskal–Wallis test was performed to determine the significance of the differences in means among groups (photosynthetic types plus ploidy levels). To test regression relationships, phylogenetic generalised least squares (PGLS) models were applied in R using the caper package (Orme *et al*., 2013; Alenazi *et al*., 2023). Values of Pagel's *λ* from zero to one represented the strength of the phylogenetic signal. The correlation coefficients (adjusted *r* ^2^) and statistical significances of covariates between variables (*P* < 0.05) were shown in the summary function in R. To summarise climatic variables in C_3_, C_3_–C_4_ intermediates, and C_4_ populations of *A. semialata*, principal component analysis (PCA) was applied with the *prcomp* function in R. The first principal component (PC1) extracted from PCA was used as an explanatory parameter in PGLS models.

## Results

### Altered hydraulic characteristics during the diversification of *Alloteropsis semialata*

**Fig. 1:**
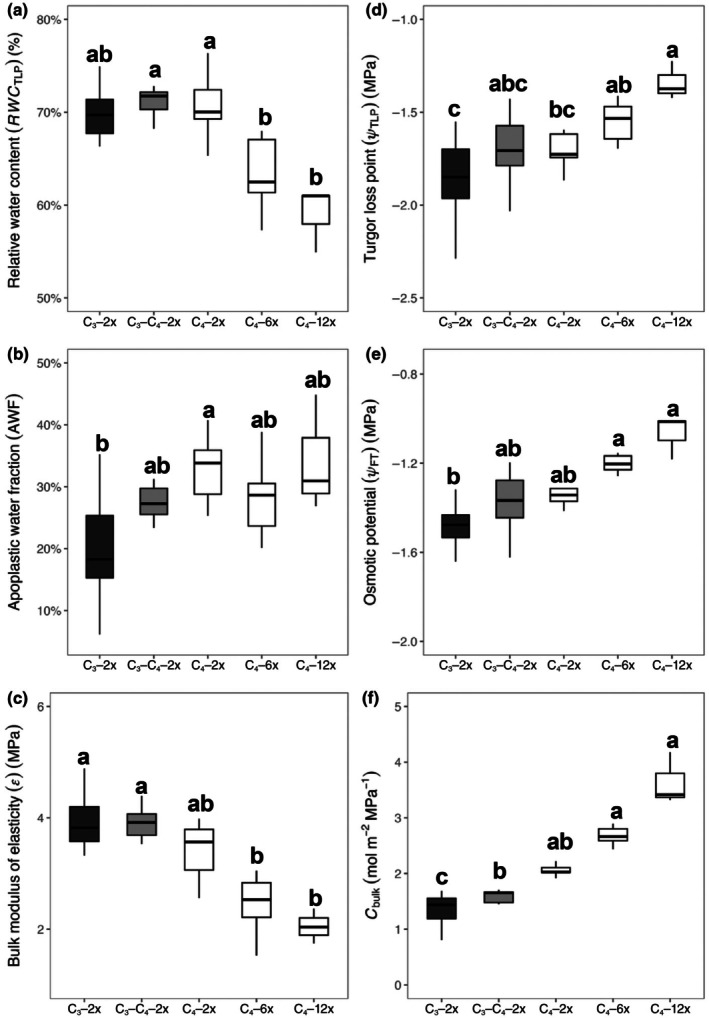
Parameters describing plant–water relations in *Alloteropsis semialata* plants differing in photosynthetic type (C_3_, C_3_–C_4_ or C_4_) and ploidy level (2x, 6x or 12x). (a) Relative water content at turgor loss point (RWC_TLP_). (b) Apoplastic water fraction at full turgor (AWF). (c) Bulk modulus of elasticity (*ε*); (d) Turgor loss point (*Ψ*_TLP_). (e) Osmotic potential at full turgor (*Ψ*_FT_). (f) Leaf capacitance (*C*_bulk_). C_3_–2x (*n* = 9, from three genotypes with three measurements each), C_3_–C_4_–2x (*n* = 9, from three genotypes with three measurements each), C_4_–2x (*n* = 9, from three genotypes with three measurements each), C_4_–6x (*n* = 9, from three genotypes with three measurements each), and C_4_–12x (*n* = 3, from one genotype with three measurements). In the boxplots, the horizontal line indicates the median, the box represents the interquartile range (IQR), and whiskers extend to 1.5 × IQR. Variables with significant group effects were subjected to a Kruskal–Wallis test, and groups that differed significantly were denoted with different letters (*P* < 0.05).

We analysed hydraulic parameters that were extracted and calculated from PV curves (Fig. S3), including RWC at the turgor loss point (RWC_TLP_), apoplastic water fraction (AWF) at full turgor, the bulk modulus of elasticity (*ε*), turgor loss point (*Ψ*_TLP_), the osmotic potential at full turgor (*Ψ*_FT_), and leaf capacitance (*C*_bulk_; Figs 1, S4–S6). As shown by Fig. 1, *A. semialata* grasses with different photosynthetic types and ploidy levels varied greatly in their water relations. When controlling for the ploidy level by comparing diploids only, AWF (Fig. 1b) and *C*_bulk_ (Fig. 1f) in C_4_ grasses were significantly higher (*P* < 0.05) than those in C_3_ plants, with C_3_–C_4_ plants having intermediate AWF values (Fig. 1b). Values of *C*_bulk_ in C_3_–C_4_ intermediates were also significantly (*P* < 0.05) greater than those in C_3_ diploids (Figs 1f, S5). However, there were no statistically significant differences in RWC_TLP_, *ε*, *Ψ*_TLP_, and *Ψ*_FT_ among diploid populations with different photosynthetic types (Fig. 1a,c–e).

Within the C_4_ populations, *RWC*_TLP_ was significantly higher (*P* < 0.05) in C_4_ diploids than in hexaploids and dodecaploids (Fig. 1a). However, C_4_ diploids had a significantly (*P* < 0.05) more negative *Ψ*_TLP_ than C_4_ dodecaploids (Figs 1d, S6). The values of AWF, *ε*, *Ψ*_FT_, and *C*_bulk_ did not differ among C_4_ diploids, hexaploids, and dodecaploids (Fig. 1b,c,e,f).

### Relationships between leaf anatomy, hydraulics, and photosynthetic characteristics

**Fig. 2:**
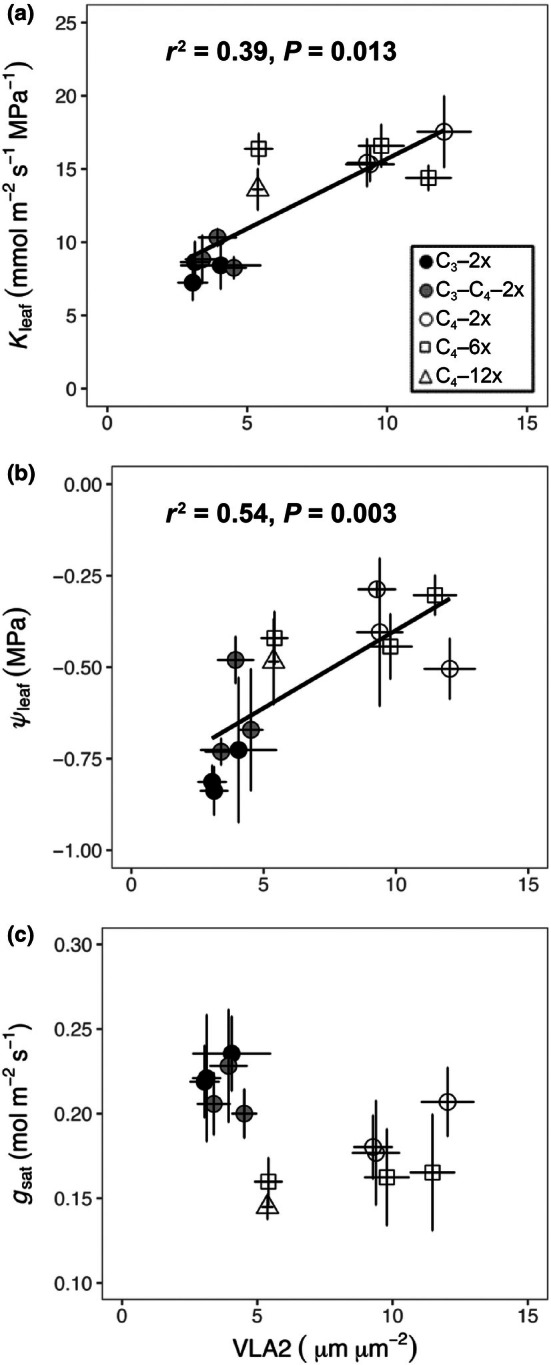
Relationships between minor vein density (VLA2) and hydraulics and stomatal conductance, including VLA2 and leaf hydraulic conductance (*K*_leaf_) (a); VLA2 and leaf water potential (*Ψ*_leaf_) (b); VLA2 and light‐saturated stomatal conductance (*g*_sat_) (c) in C_3_ grasses (black circles), C_3_–C_4_ grasses (grey circles), C_4_–2x grasses (white circles), C_4_–6x grasses (white squares) and C_4_–12x grasses (white triangles) of *Alloteropsis semialata* . Each symbol represents mean values (*n* = 3). Error bars indicate means ± SEs. Phylogenetic generalised least squares (PGLS) model adjusted *r* ^2^ and *P*‐value results are shown.

**Fig. 3:**
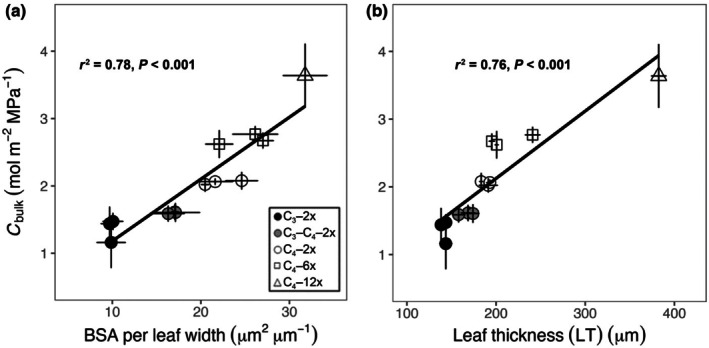
Relationships between leaf anatomy and leaf capacitance (*C*_bulk_), including the ratio of total bundle sheath area to leaf width (BSA per leaf width) and *C*_bulk_ (a); leaf thickness (LT) and *C*_bulk_ (b) in C_3_ grasses (black circles), C_3_–C_4_ grasses (grey circles), C_4_–2x grasses (white circles), C_4_–6x grasses (white squares) and C_4_–12x grasses (white triangles) of *Alloteropsis semialata* . Each symbol represents mean values (*n* = 3). Error bars indicate means ± SEs. Phylogenetic generalised least squares (PGLS) model adjusted *r* ^2^ and *P*‐value results are shown.

**Fig. 4:**
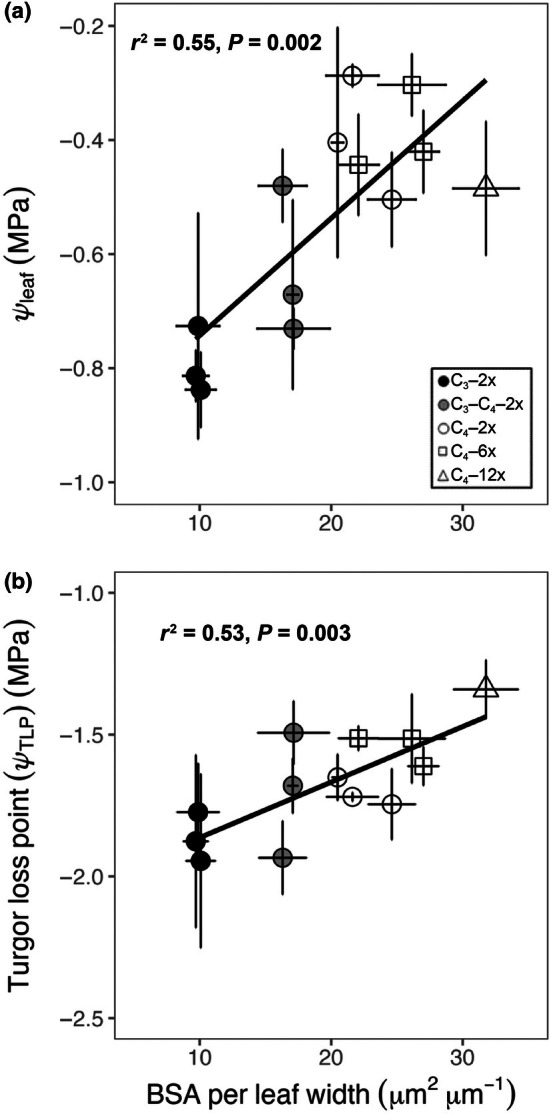
Relationships between the ratio of total bundle sheath area to leaf width (BSA per leaf width) and leaf hydraulics, including BSA per leaf width and the leaf water potential (*Ψ*_leaf_) (a); BSA per leaf width and turgor loss point (*Ψ*_TLP_) (b) in C_3_ grasses (black circles), C_3_–C_4_ grasses (grey circles), C_4_–2x grasses (white circles), C_4_–6x grasses (white squares) and C_4_–12x grasses (white triangles) of *Alloteropsis semialata* . Each symbol represents mean values (*n* = 3). Error bars indicate means ± SEs. Phylogenetic generalised least squares (PGLS) model adjusted *r* ^2^ and *P*‐value results are shown.

We explored the interactions between anatomical features and leaf hydraulics across C_3_ diploids, C_3_–C_4_ diploids, C_4_ diploids, C_4_ hexaploids, and C_4_ dodecaploids (Fig. S7), finding that plant–water relations were related to leaf anatomy (Figs 2, 3, 4, S7). Specifically, minor vein density (VLA2) was significantly associated with both leaf hydraulic conductance (*K*_leaf_) and leaf water potential (*Ψ*_leaf_) across the groups (Fig. 2a,b). However, VLA2 was unrelated to stomatal conductance (*g*_s_; Fig. 2c). In Fig. 3, the increased BSA per leaf width was significantly associated with higher *C*_bulk_ in all grasses (Fig. 3a). This was consistent with Fig. S8, LT increased after polyploid formation and was also positively associated with *C*_bulk_, such that LT contributed to *C*_bulk_ across all grasses (Fig. 3b). In combination, BSA per leaf width and LT had additive effects accounting for 84% of the variation in *C*_bulk_ (Fig. S9). LT showed a stronger phylogenetic signal than BSA per leaf width.

**Fig. 5:**
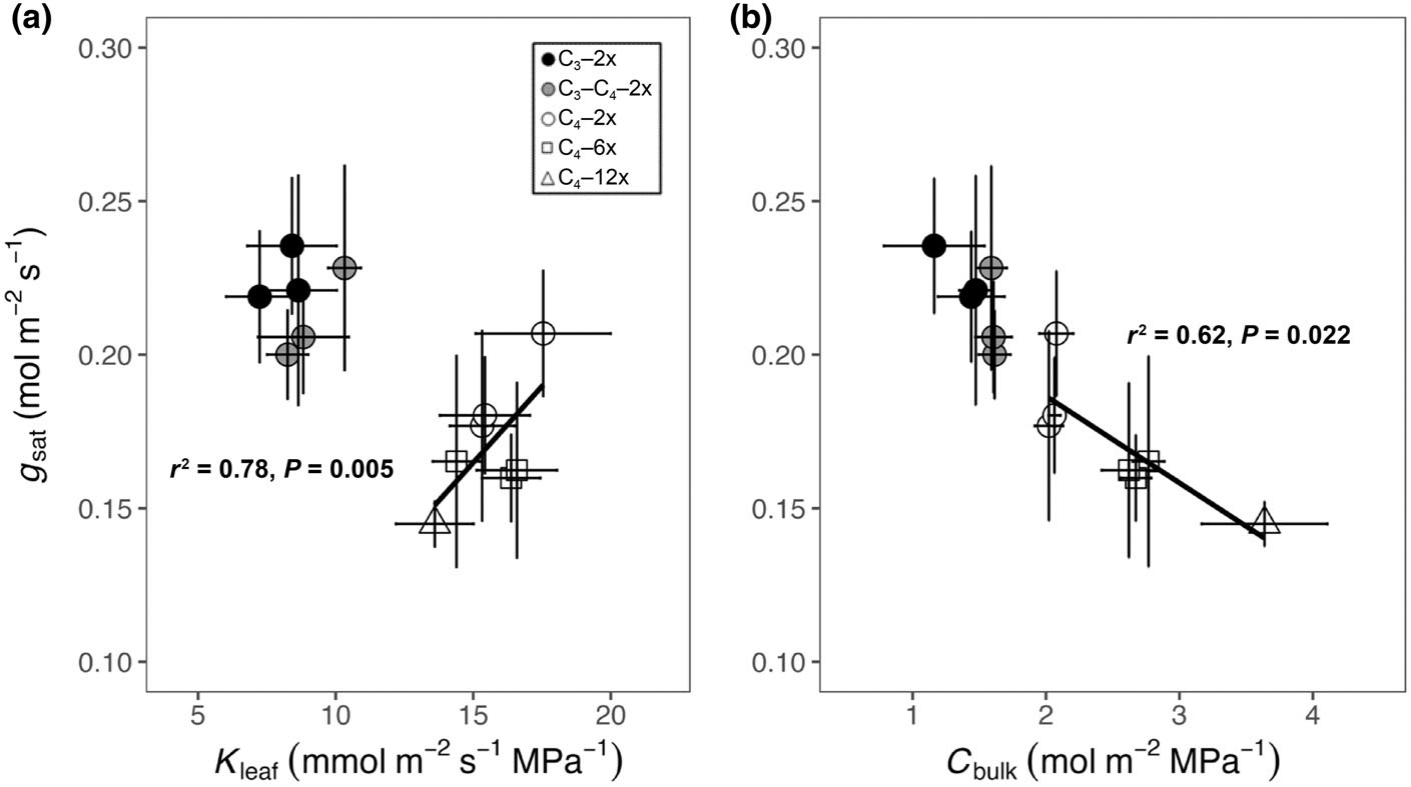
Relationships between leaf hydraulics and the light‐saturated stomatal conductance (*g*_sat_), including leaf hydraulic conductance (*K*_leaf_) and *g*_sat_ (a); leaf capacitance (*C*_bulk_) and *g*_sat_ (b) in C_3_ grasses (black circles), C_3_–C_4_ grasses (grey circles), C_4_–2x grasses (white circles), C_4_–6x grasses (white squares) and C_4_–12x grasses (white triangles) of *Alloteropsis semialata* . Each symbol represents mean values (*n* = 3). Error bars indicate means ± SEs. Phylogenetic generalised least squares (PGLS) model adjusted *r* ^2^ and *P*‐value results are shown.

The BSA per leaf width was also related significantly to *Ψ*_leaf_ among all populations (Fig. 4a), such that higher values of BSA per leaf width were associated with less negative *Ψ*_leaf_. Similarly, higher values of BSA per leaf width were associated with less negative *Ψ*_TLP_ (Fig. 4b). Compared with C_3_ and C_3_–C_4_ populations, C_4_ grasses had a distinct positioning in trait space (*K*_leaf_ and *g*_sat_) because of the combination of relatively high *K*_leaf_ and lower *g*_s_, which was consistent with the study from Baird *et al*. (2025). In addition, the results of PGLS modelling showed both *K*_leaf_ and photosynthetic types were significantly related to *g*_sat_. Therefore, the analysis of relationships between leaf hydraulic traits and *g*_sat_ were separated into two groups by photosynthetic types in Fig. 5. Across non‐C_4_ populations, there was no significant relationship between *K*_leaf_ and *g*_sat_ (Fig. 5a), which may be partly because of the smaller sample sizes. However, *K*_leaf_ was significantly associated with the *g*_sat_ among C_4_ populations (Fig. 5a), suggesting that higher *K*_leaf_ may contribute to higher *g*_sat_ in C_4_ accessions. It was interesting to note that *C*_bulk_ was also associated with *g*_sat_ (Fig. 5b), although in the opposite direction compared with *K*_leaf_, such that higher *C*_bulk_ was related to lower *g*_sat_ in C_4_ populations (Fig. 5b).

### Links between leaf anatomy, hydraulics, and climate data

**Fig. 6:**
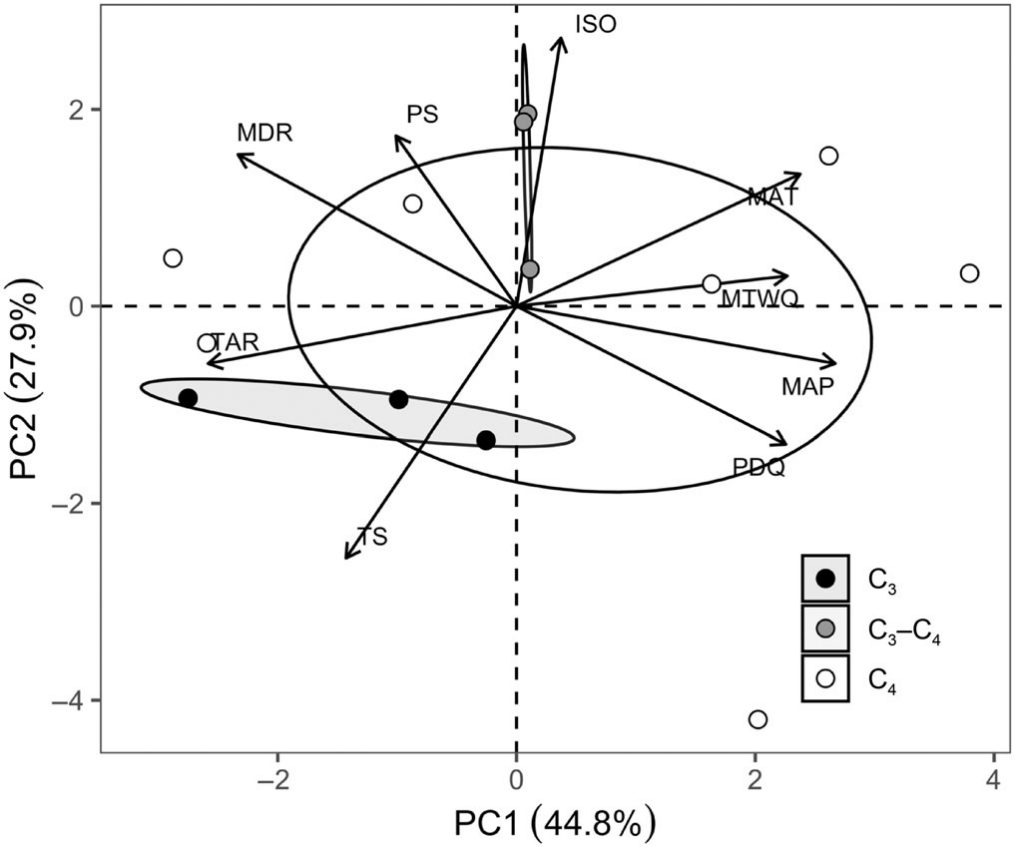
Principal component analysis (PCA) of climatic parameters in C_3_ diploid grasses (black circles), C_3_–C_4_ diploid grasses (grey circles) and C_4_ diploid grasses (open circles) of *Alloteropsis semialata* . Each symbol represents mean values (*n* = 3). The populations are represented in the space defined by the first two principal components PC1 and PC2. Black arrows in the same space represent different climatic variables. Abbreviations are as follows: mean annual temperature (MAT), mean annual precipitation (MAP), mean temperature of wettest quarter (MTWQ), precipitation seasonality (PS), temperature seasonality (TS), precipitation of driest quarter (PDQ), mean diurnal range (MDR), temperature annual range (TAR), and isothermality (ISO).

**Fig. 7:**
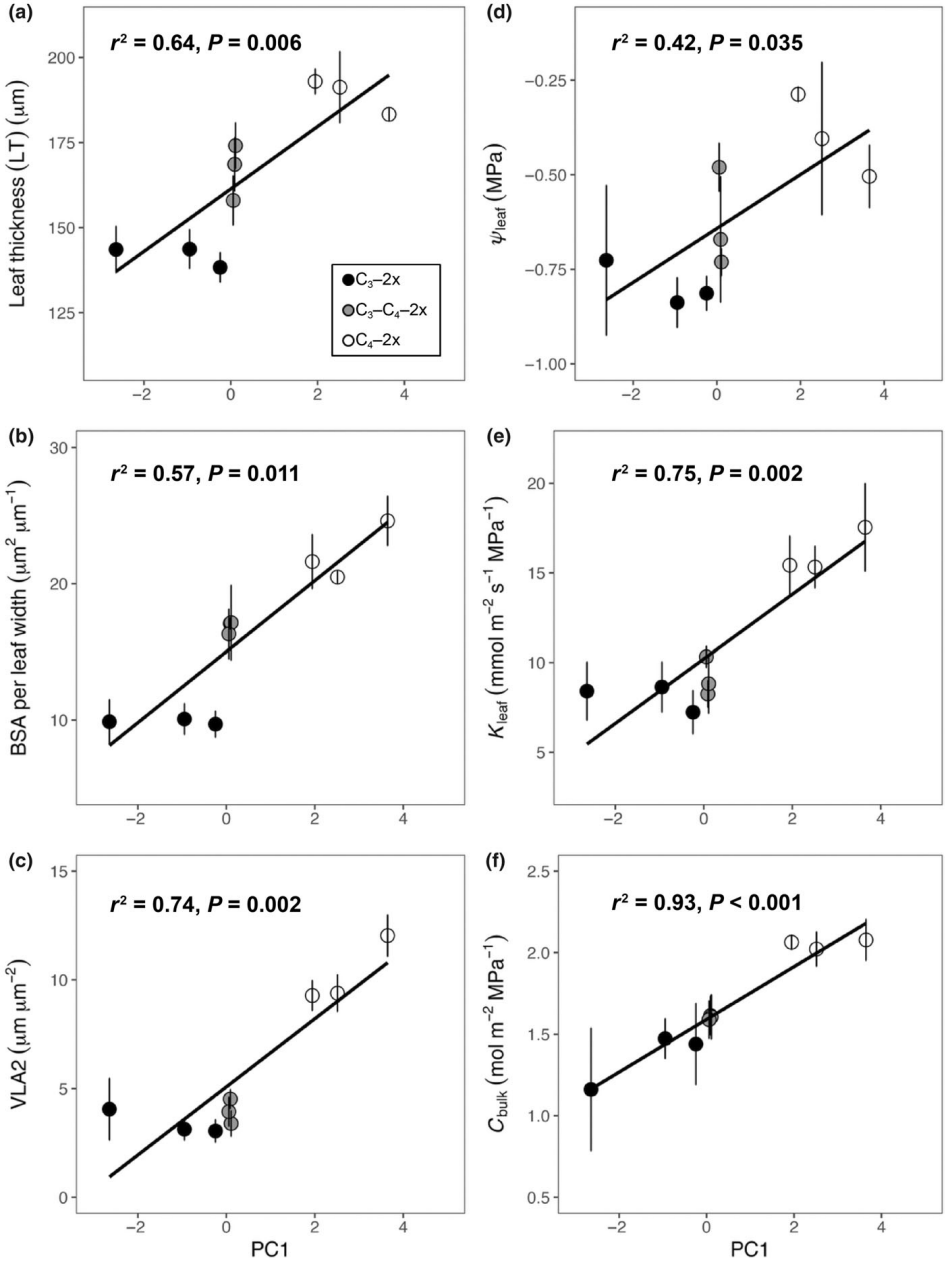
Relationships between first principal component (PC1) and leaf anatomy or leaf hydraulics, including leaf thickness (a), total bundle sheath area to leaf width ratio (BSA per leaf width) (b), and minor vein density (VLA2) (c); relationships between PC1 and leaf water potential (*Ψ*_leaf_) (d), leaf hydraulic conductance (*K*_leaf_) (e), and leaf capacitance (*C*_bulk_) (f) in C_3_ grasses (black circles), C_3_–C_4_ grasses (grey circles), C_4_–2x grasses (white circles), C_4_–6x grasses (white squares) and C_4_–12x grasses (white triangles) of *Alloteropsis semialata* . Each symbol represents mean values (*n* = 3). Error bars indicate means ± SEs. Phylogenetic generalised least squares (PGLS) model adjusted *r* ^2^ and *P*‐value results are shown.

A PCA was first applied to identify the broad climatic niche of *A. semialata* (Fig. 6) since temperature and precipitation could be significantly related to one another (Bone *et al*., 2015; Leverett *et al*., 2021). The PC1 parameter (explaining 44.8% of the variance; Fig. 6) was strongly related to several climate factors related to both temperature and precipitation. In particular, mean annual temperature (MAT), mean temperature of wettest quarter (MTWQ), mean annual precipitation (MAP), and precipitation of driest quarter (PDQ) had positive loadings on PC1, while temperature seasonality (TS), precipitation seasonality (PS), mean diurnal range (MDR) and temperature annual range (TAR) had negative loadings on PC1. Thus, temperature, precipitation and seasonality covaried across the geographical ranges of both C_4_ and non‐C_4_ diploid populations. Moreover, C_4_ grasses clearly have a larger climate niche than C_3_ grasses (Fig. 6), consistent with past observations for this species (Lundgren *et al*., 2015). Although PC1 explained the largest proportion of total climatic variance, separation between C_3_ and C_4_ populations was more evident along PC2 (Fig. 6). Most C_4_ populations were positioned towards one end of PC2, with two exceptions, whereas C_3_ populations occupied contrasting values. In addition, there were no significant relationships between PC1 and leaf anatomical and hydraulic traits (Fig. S10). However, when the picture was simplified by excluding higher ploidy levels in C_4_ grasses, PC1 was significantly associated with anatomical features and hydraulic characteristics across all diploid populations (Fig. 7). Specifically, PC1 was significantly positively related to LT, BSA per leaf width, and VLA2 in diploid populations of non‐C_4_ and C_4_ plants (Fig. 7a–c), indicating C_4_ diploid populations with higher LT, bundle sheath tissue, and VLA2 are adapted to hotter, wetter, less seasonal environments. PC1 was also positively associated with *Ψ*_leaf_, *K*_leaf_, and *C*_bulk_ in non‐C_4_ and C_4_ diploid populations (Fig. 7d–f), suggesting that higher hydraulic capacity in C_4_ diploid species may adapt them to hotter, wetter, and less seasonal habitats.

## Discussion

The grass *Alloteropsis semialata* shows a high level of intraspecific variation in photosynthesis, including C_3_, C_4_ and C_3_–C_4_ intermediate forms (Lundgren *et al*., 2016; Alenazi *et al*., 2024; Zhou & Osborne, 2024) and ploidy (Bianconi *et al*., 2020), which makes it an excellent model for understanding the relationships between leaf structure and function. Our results support the hypothesis that the leaf anatomy required for C_4_ photosynthesis can improve the hydraulic system. We also find that environmental adaptation is an additional reason for the more efficient hydraulic system in C_4_ diploid plants than in C_3_ and C_3_–C_4_ plants of this species.

Higher leaf hydraulic conductance and leaf capacitance in C_4_ *A. semialata* may limit exposure to leaf water deficits by aiding the supply and buffering of leaf water, respectively. Strong sensitivity of photosynthesis in wild C_4_ grasses to severe drought conditions has been reported in previous work (Ghannoum *et al*., 2003; Ripley *et al*., 2007). Improved leaf hydraulics may therefore contribute to the high performance of photosynthesis in wild C_4_ grasses in hot and dry environmental conditions (Osborne & Freckleton, 2009; Watcharamongkol *et al*., 2018). Minor vein density (VAL2) increased during the evolution of C_4_ photosynthesis in *A. semialata* through the insertion of an additional order of veins (5° veins, Lundgren *et al*., 2016), increasing the total bundle sheath area to leaf width ratio (BSA per leaf width) in comparison with C_3_–C_4_ intermediates. This followed the bundle sheath tissue enlargement that occurred in C_3_–C_4_ intermediates compared with the C_3_ type (Lundgren *et al*., 2016; Alenazi *et al*., 2023). C_3_ populations with fewer minor veins and a smaller area of bundle sheath tissues have lower hydraulic conductance and leaf capacitance, thereby increasing exposure to leaf water deficits compared with the C_4_ type. These findings support our hypothesis that larger bundle sheath tissues are associated with higher leaf capacitance, and higher vein densities are linked to greater leaf hydraulic conductance in C_4_ compared with C_3_ plants.

Environmental factors also played a key role in the variations in leaf structure and hydraulic function in the warm, dry, grassland, savanna, and woodland habitats where *A. semialata* grows. In this study, hotter, wetter, and less seasonal climatic conditions were positively associated with LT, BSA per leaf width, VLA2, *Ψ*_leaf_, *K*_leaf_, and *C*_bulk_ in non‐C_4_ and C_4_ diploid populations, suggesting enhanced leaf capacitance and hydraulic conductance are adaptations to warmer, wetter, and less seasonal habitats. Thus, both leaf structural features associated with Kranz anatomy and environmental variables significantly contributed to C_4_ diploid plants having a higher hydraulic capacity. The diversity in hydraulic performance in *A. semialata* further improves our understanding of how C_4_ plants coordinate anatomical and hydraulic traits to survive in hot environments.

### Distinct leaf structure in C_4_ plants promotes higher leaf hydraulic conductance and capacitance than in C_3_ species

Based on the combination of phylogenetic comparative and physiological analysis, we found that modifications of leaf anatomy affect leaf physiological function, especially in C_4_ grasses (Figs 2, 3, 4, 5, 6). Our work provides empirical support for previous theoretical work (Osborne & Sack, 2012; Griffiths *et al*., 2013), arguing that higher minor vein density should be associated with greater leaf hydraulic conductance and less negative leaf water potential (Fig. 2a,b). A larger number of minor veins, more closely spaced, could raise hydraulic conductance by increasing the total number of xylem vessels within the leaf (i.e. leaf xylem hydraulic conductance, *K*_x_) or by reducing the path length for water flow from the xylem to sites of evaporation (i.e. the component of *K*_leaf_ outside the xylem, *K*_ox_). An emerging consensus shows that *K*_ox_ exerts greater control than *K*_x_ over *K*_leaf_ (Scoffoni *et al*., 2023). However, a recent comparison of hydraulics between C_3_ and C_4_ grasses found differences in *K*_x_ but not *K*_ox_ (Baird *et al*., 2025). Previous studies have proposed that water relations are central to the evolution of the C_4_ photosynthetic pathway (Kocacinar & Sage, 2003; Kocacinar *et al*., 2008; Osborne & Sack, 2012; Ocheltree *et al*., 2014) and may explain the selection for modifications in leaf anatomy during the early evolution of C_4_ photosynthesis. Moreover, the fact that these changes are found in other lineages of C_3_, C_3_–C_4_ intermediates and CAM species (Osborne & Sack, 2012) suggests that they are neither rare nor exceptional.

Reduced interveinal distance and enlarged bundle sheath tissues, in theory, are key preconditions required for the evolution of C_4_ photosynthesis (Christin *et al*., 2013; Griffiths *et al*., 2013; Zhou *et al*., 2018). However, different modifications made to meet the same requirements of C_4_ photosynthesis may lead to different functional consequences in each C_4_ lineage (Lundgren *et al*., 2014). For example, C_4_ *Axonopus compressus* evolved thin leaves with small cells and short interveinal distances, while C_4_ *Alloteropsis cimicina* developed thick leaves with large bundle sheath tissues (Lundgren *et al*., 2014), similar to our result that C_4_ *A. semialata* evolved a higher LT than its C_3_ relatives (Fig. 3b). We find that higher values of leaf capacitance are not only related to a greater ratio of bundle sheath area : leaf width, as predicted from theory (Sage, 2004), but are also associated with LT in plants with varying ploidy levels. This is consistent with previous work observed among other plant taxa (Sack *et al*., 2003), especially in relation to succulence (Leverett *et al*., 2023). These associations of hydraulic traits with numerous small veins, bundle sheath traits, and LT among C_3_ and C_4_ grasses provide new insights into the functional consequences of C_4_ evolution. Our findings suggest that selection for greater leaf capacitance is a plausible functional mechanism to explain how an enlarged bundle sheath evolves first in C_3_ (Christin *et al*., 2013) and then in C_3_–C_4_ intermediates as an anatomical precursor for the evolution of the C_4_ CCM (Sage, 2001, 2004; Osborne & Sack, 2012; Griffiths *et al*., 2013; Zhou *et al*., 2025). Thus, C_4_ evolution involves coordinated changes in leaf anatomy, including increased vein density and modified bundle sheath structure. These anatomical shifts may influence leaf water storage capacity and thus capacitance.

On the contrary, although we did not quantify suberisation in bundle sheath tissues, variation in suberin deposition could influence radial water movement and hydraulic conductance in *A. semialata*. Future work assessing the extent of suberisation in these populations would help clarify its potential role. Among C_3_ and C_4_ plants, the diversity of leaf hydraulics could be driven by variation in the suberin sheath properties (Baird *et al*., 2025) since a thicker and more continuous suberin sheath could reduce water flow from veins to stomata, leading to a lower *K*_leaf_. For instance, previous work reported that an expanded vein sheath perimeter provides more surface area for exchange with the surrounding mesophyll via both the symplast and apoplast (Baird *et al*., 2025). This facilitates more water movement through membrane aquaporins, plasmodesmata, and cell wall transport pathways while overcoming suberin and lignin barriers, and could enhance hydraulic conductance (Mertz & Brutnell, 2014; Sade *et al*., 2015; Baird *et al*., 2025). In addition, the diversity of suberin deposition, such as variations in thickness, continuity, and chemical composition across C_4_ *A. semialata*, may also represent secondary adaptation to hot, wet and less seasonal environments. For example, reduced suberisation might occur in wetter environments to allow higher water movements, while enhanced suberisation could evolve under drier or more saline conditions to conserve water. Our results show C_4_ plants have higher *K*_leaf_ and *C*_bulk_ in wetter environments (Fig. 7e,f), which may be associated with the thinner suberin sheath. However, more work is required to identify how variation in suberin sheath impacts leaf hydraulics in *A. semialata*.

### Environmental factors influence variations in anatomical and hydraulic characteristics

Environmental conditions such as high light and temperature, low CO_2_, salinity, and drought have been widely proposed as facilitators for the C_4_ photosynthetic pathway (Sage & Pearcy, 2000; Sage, 2001; Osborne & Freckleton, 2009; Osborne & Sack, 2012; Watcharamongkol *et al*., 2018). Arid and/or saline conditions in combination with open habitat availability enhance the competitiveness of C_4_ over C_3_ species (Osmond *et al*., 1982; Sage & Pearcy, 2000; Sage, 2001). After C_4_ evolution, polyploid formation has occurred in *A. semialata* during the diversification of C_4_ lineages (Lundgren *et al*., 2015; Bianconi *et al*., 2020), leading to increases in cell size (Lundgren *et al*., 2019b; Zhou & Osborne, 2024). Leaf structures arising from varied cell size are often tightly linked to physiological functions (Brodribb *et al*., 2013). For example, smaller cell size contributes to high vein density and stomatal density, which further builds a highly efficient leaf vascular system to enable rapid water transport (Brodribb *et al*., 2013). But do hot and dry habitats or polyploid formation also favour anatomical and hydraulic traits and their interactions during the evolutionary history of C_4_ grasses?

We found that a series of shifts in leaf anatomy and hydraulic traits were caused by the formation of polyploids, including hexaploids and dodecaploids, during C_4_ diversification (Figs 1, S4–S9, S11–S15). This shows that polyploid formation in C_4_ grasses may increase the diversity of anatomical and hydraulic characteristics. Polyploidy in this species is associated with a range of anatomical traits, including increased LT, mesophyll cell size, bundle sheath cell size, epidermis cell size, bulliform cell size, and decreased average distance between major veins (Lundgren *et al*., 2019b), which may further contribute to enhanced leaf hydraulic conductance and dehydration tolerance in C_4_ progenitors (Sage, 2004; Osborne & Sack, 2012; Griffiths *et al*., 2013).

Climate adaptation was also important. For example, locally warmer and drier climate habitats are linked to stronger activity of the C_4_ cycle (Alenazi *et al*., 2023). Our analysis shows PC1 is positively associated with LT, BSA per leaf width, VLA2, *Ψ*_leaf_, *K*_leaf_, and *C*_bulk_ (Fig. 7), indicating that selection for anatomical traits by hotter, wetter and less seasonal environments in C_4_ diploid grasses may also contribute to hydraulic trait values. This shows that the effect of ecological factors is multifaceted. Local adaptation to climatic variables potentially explains some of the diversity of leaf anatomy (Figs S11, S13–S15) and physiology (Figs S4–S6, S12) in *A. semialata*, that is the relationships between leaf physiology and habitats may be a mechanism that selects for more C_4_‐like phenotypes. Unfortunately, there are not many studies on the influence of ploidy or its interactions with environmental conditions on hydraulics and leaf anatomy. Accordingly, our results provide insights into how anatomical and hydraulic traits diversify as secondary adaptations to novel combinations of environments and polyploid formation.

### Higher hydraulic capacity in C_4_ plants as an adaptation to hotter, wetter and less seasonal environments

One of the major environmental factors limiting water transport and photosynthesis is plant–water deficit caused by high atmospheric VPD (or high temperature) or soil drying (Ghannoum, 2009; Flexas *et al*., 2018; Xiong & Nadal, 2020). Besides water loss through stomata and water flow through the leaf constrained by *K*_leaf_, hydraulic capacitance is considered the third central characteristic determining the overall water potential of the leaf (Xiong & Nadal, 2020). The higher hydraulic capacity (higher *K*_leaf_ and *C*_bulk_) in C_4_ than C_3_ plants in this study (Fig. 1) is significantly positively related to hotter, wetter, and less seasonal environments (Fig. 7). How might this represent an environmental adaptation?

Plants usually reach high transpiration rates under hot and high VPD conditions, increasing water loss from leaves (Sadok *et al*., 2021). To maintain both leaf photosynthetic capacity and leaf cooling, plants require a rapid and continuous water supply to leaves. Thus, a high hydraulic capacity could support plants in maintaining an adequate water supply under high evaporative conditions, minimising the risk of leaf dehydration (Brodribb *et al*., 2007; Sage *et al*., 2018). C_4_ plants with higher minor vein density and larger bundle sheath tissues could meet this high evaporative demand (Sage *et al*., 2018), since more minor veins could enhance water flow to individual cells (Sage, 2004; Osborne & Sack, 2012; Griffiths *et al*., 2013; Sage *et al*., 2018) and enlarged bundle sheath tissues may play a role as the hydraulic buffer to supply water during leaf transpiration (Sage, 2004; Osborne & Sack, 2012; Sage *et al*., 2018). This may also reduce the risk of cavitation in vascular tissues under high and fluctuating transpiration demand (Sage, 2004; Osborne & Sack, 2012; Sage *et al*., 2018). Additionally, the recently described nonstomatal control of transpiration is a leaf hydraulic property in mesophyll that regulates the resistance to movement of water across cell membranes (Márquez *et al*., 2024). When leaves in C_4_ plants are exposed to increased atmospheric VPD and nonstomatal control exceeds the stomatal resistance, this mechanism leads to incomplete saturation of leaf–air spaces, contributing to reducing water loss and maintaining photosynthesis (Márquez *et al*., 2024).

Previous work has proposed that the capacity of the hydraulic system seems to determine the maximum leaf photosynthetic potential (Scoffoni *et al*., 2016; Xiong & Nadal, 2020). In wet environments, plants may operate a high‐water consumption strategy because abundant water availability makes hydraulic efficiency an asset rather than a cost. Therefore, an enhanced hydraulic system in plants can optimise photosynthetic carbon assimilation when water limitations are not experienced, which is consistent with a previous study showing that lianas appeared to exhibit enhanced photosynthetic performance in the wet season (Chen *et al*., 2015). Similarly, plants can grow continuously throughout the year in less seasonal environments, such as tropical rainforests, equatorial lowlands, and coastal wet tropics. To support overall plant productivity in stable climates, plants demand consistent water transport and photosynthesis, which underscores the importance of a well‐developed hydraulic system in sustaining physiological performance over time.

### Conclusions

We linked leaf physiology and anatomy to understand the evolution of hydraulic coordination among C_3_, C_3_–C_4_ and C_4_ grasses in relation to anatomical structure and function. Higher leaf hydraulic conductance and water potential in C_4_ leaves are associated with larger minor vein density, while a greater ratio of bundle sheath area : leaf width and thicker leaves contribute to higher values of leaf capacitance in C_4_ plants. These findings are consistent with the hypothesis that anatomical adaptations for C_4_ photosynthesis also improve plant–water relations. Polyploid formation and changes in climate niche during the evolution of C_4_ photosynthesis also explain variations in leaf anatomy and water relations. Both leaf anatomy and environmental adaptation could therefore enhance the hydraulic system during C_4_ evolution. C_4_ photosynthesis expands the hydrological niche in *A. semialata*, and the traits highlighted here explain how C_4_ plants adapt to a broad range of habitats.

## Competing interests

None declared.

## Author contributions

YZ and CPO conceived and designed the study. YZ collected and analysed the data and produced a first draft of the manuscript. LTD, HL and CPO reviewed the manuscript and provided comments and feedback. All authors critically contributed to the manuscript and gave final approval for publication.

## Disclaimer

The New Phytologist Foundation remains neutral with regard to jurisdictional claims in maps and in any institutional affiliations.

## Supporting information


**Fig. S1** Comparison between the bundle sheath tissue and leaf width between the first cut of leaf cross‐section and the last cut of leaf cross‐section among all individuals.
**Fig. S2** Diagram of the relationship between photosynthetic photon flux density and stomatal conductance.
**Fig. S3** Diagram of the hydraulic traits.
**Fig. S4** Ancestral state reconstruction of bulk modulus elasticity (*ε*) in *A. semialata* .
**Fig. S5** Ancestral state reconstruction of leaf capacitance (*C*_bulk_) in *A. semialata* .
**Fig. S6** Ancestral state reconstruction of turgor loss point (*Ψ*_TLP_) in *A. semialata* .
**Fig. S7** Relationships between leaf anatomy and leaf hydraulics.
**Fig. S8** Relationship between bundle sheath cell and leaf hydraulics.
**Fig. S9** Interactive effect of total bundle sheath area to leaf width ratio (BSA per leaf width) and leaf thickness on leaf capacitance (*C*_bulk_) in *A. semialata* .
**Fig. S10** Relationships between PC1 and leaf anatomy or hydraulics.
**Fig. S11** Leaf cross‐sections representing 13 populations of *A. semialata* .
**Fig. S12** Ancestral state reconstruction of leaf hydraulic conductance (*K*_leaf_) in *A. semialata* .
**Fig. S13** Ancestral state reconstruction of leaf thickness in *A. semialata* .
**Fig. S14** Ancestral state reconstruction of the total bundle sheath area to leaf width ratio (BSA per leaf width) in *A. semialata* .
**Fig. S15** Ancestral state reconstruction of minor leaf vein density (VLA2) in *A. semialata* .
**Table S1** Details of the *Alloteropsis semialata* populations used in this study.
Please note: Wiley is not responsible for the content or functionality of any Supporting Information supplied by the authors. Any queries (other than missing material) should be directed to the *New Phytologist* Central Office.

## Data Availability

The data that support the findings of this study are openly available in Zenodo at https://doi.org/10.5281/zenodo.19448359.
