# Supplementary material for: C4 photosynthetic anatomy is associated with higher leaf hydraulic conductance and capacitance in Alloteropsis semialata
Source: New Phytol. 2026 Apr 29;250(6):3687–700. doi: 10.1111/nph.71221 (PMC13193344; doi:10.1111/nph.71221)
Supplement: Supplementary file 1 — Fig. S1 Comparison between the bundle sheath tissue and leaf width between the first cut of leaf cross‐section and the last cut of leaf cross‐section among all individuals. Fig. S2 Diagram of the relationship between photosynthetic photon flux density and stomatal conductance. Fig. S3 Diagram of the hydraulic traits. Fig. S4 Ancestral state reconstruction of bulk modulus elasticity (ε) in A. semialata . Fig. S5 Ancestral state reconstruction of leaf capacitance (C bulk) in A. semialata . Fig. S6 Ancestral state reconstruction of turgor loss point (Ψ TLP) in A. semialata . Fig. S7 Relationships between leaf anatomy and leaf hydraulics. Fig. S8 Relationship between bundle sheath cell and leaf hydraulics. Fig. S9 Interactive effect of total bundle sheath area to leaf width ratio (BSA per leaf width) and leaf thickness on leaf capacitance (C bulk) in A. semialata . Fig. S10 Relationships between PC1 and leaf anatomy or hydraulics. Fig. S11 Leaf cross‐sections representing 13 populations of A. semialata . Fig. S12 Ancestral state reconstruction of leaf hydraulic conductance (K leaf) in A. semialata . Fig. S13 Ancestral state reconstruction of leaf thickness in A. semialata . Fig. S14 Ancestral state reconstruction of the total bundle sheath area to leaf width ratio (BSA per leaf width) in A. semialata . Fig. S15 Ancestral state reconstruction of minor leaf vein density (VLA2) in A. semialata . Table S1 Details of the Alloteropsis semialata populations used in this study. Please note: Wiley is not responsible for the content or functionality of any Supporting Information supplied by the authors. Any queries (other than missing material) should be directed to the New Phytologist Central Office. [file NPH-250-3687-s001.pdf]

New Phytologist **Supporting Information**

Article title: C_4 photosynthetic anatomy is associated with higher leaf hydraulic conductance and capacitance in *Alloteropsis semialata* (R. Br.) Hitchc.

Authors: Yanmin Zhou (周艳敏), Luke T. Dunning, Hui Liu (刘慧), Colin P. Osborne

Article acceptance date: 31 March 2026

Supporting information

Tab. S1 Details of the *A. semialata* populations used in this study, including individual, population, alternative id, country of origin, the carbon isotope composition of plant tissues ($\delta^{13}C$), $\delta^{13}C$ source, photosynthetic type, ploidy level and repeat number of each individual.

**Individual**	**Population**	**Alternative id**	**Country**	**$\delta^{13}C$ (‰)**	**$\delta^{13}C$ source**	**Photosynthetic type**	**Ploidy**	**Repeats**
EML11-200	EML11	EML-11	South Africa	-27.3	Olofsson et al. 2021	C_3	2x	3
KWT3-3	KWT3	KWT-3	South Africa	-31.2	Bianconi et al. 2020	C_3	2x	3
ZIM15-03-f2	ZIM15-03	ZIM1503-01	Zimbabwe	-26.6	Olofsson et al. 2021	C_3	2x	3
L01A	L01A	L01-A	Tanzania	-23.1	Lundgren et al. 2016	C_3–C_4	2x	3
L04B	L04B	L04-B	Tanzania	-23.4	Lundgren et al. 2016	C_3–C_4	2x	3
TAN16-02-03C	TAN16-02	TAN16-02-03	Tanzania	-25.2	Olofsson et al. 2021	C_3–C_4	2x	3
MAJ3-1	MAJ3	Maj-3	Madagascar	-11.9	Lundgren et al. 2016	C_4	2x	3
PHIL16-13B	PHIL16	PHIL16-01	Philippines	-15.2	Olofsson et al. 2021	C_4	2x	3
TWN10-2	TWN10	TW10	Taiwan	-13.9	Bianconi et al. 2020	C_4	2x	3
MDG1-2	MDG1	MDG-1	South Africa	-11.1	Lundgren et al. 2016	C_4	6x	3
ZAM15-02-01	ZAM15-02	ZAM1502-01	Zambia	-12.2	Olofsson et al. 2021	C_4	6x	3
ZAM17-27	ZAM17	ZAM1727-01	Zambia	-11.4	Olofsson et al. 2021	C_4	6x	3
CAM16-01-02	CAM16-01	CAM1601-02	Cameroon	-12.3	Lundgren et al. 2019	C_4	12x	3

Fig. S1 Comparison of the bundle sheath tissue and leaf width between the first cut of leaf cross-section and the last cut of leaf cross-section among all individuals. Specifically, (a) comparison between the first and last cuts of leaf cross-section in parenchymatous bundle sheath cell (PBSC) width; (b) comparison between the first and last cuts of leaf cross-section in inner bundle sheath cell (IBSC) width; (c) comparison between the first and last cuts of leaf cross-section in leaf width. The distance between the first cut of leaf cross-section and the last cut of leaf cross-section is 2.0-2.5 mm. Data dots are coloured and shaped by C_3–2x black circles (n=9, from three genotypes of three measurements each), C_3–C_4–2x grey circles (n=9, from three genotypes of three measurements each), C_4–2x white circles (n=9, from three genotypes of three measurements each), C_4–6x white squares (n=9, from three genotypes of three measurements each), and C_4–12x white triangles (n=3, from one genotype of three measurements).

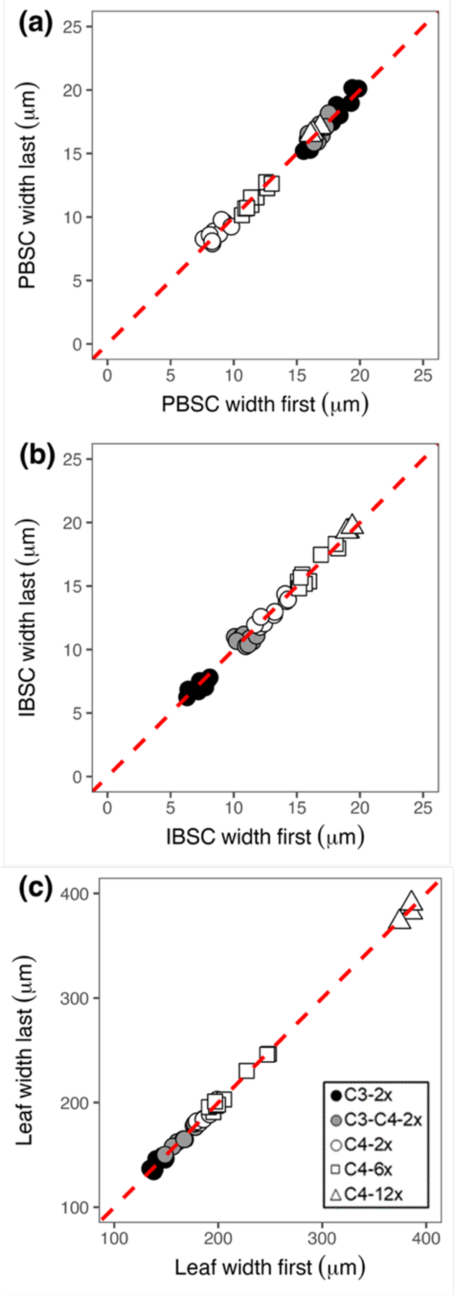

Fig. S2 Diagram of the relationship between photosynthetic photon flux density (PPFD) and stomatal conductance, including light saturation point and light-saturated stomatal conductance (g_{sat}). g_{sat} is the maximum value of stomatal conductance, where higher light intensity no longer increases stomatal conductance.

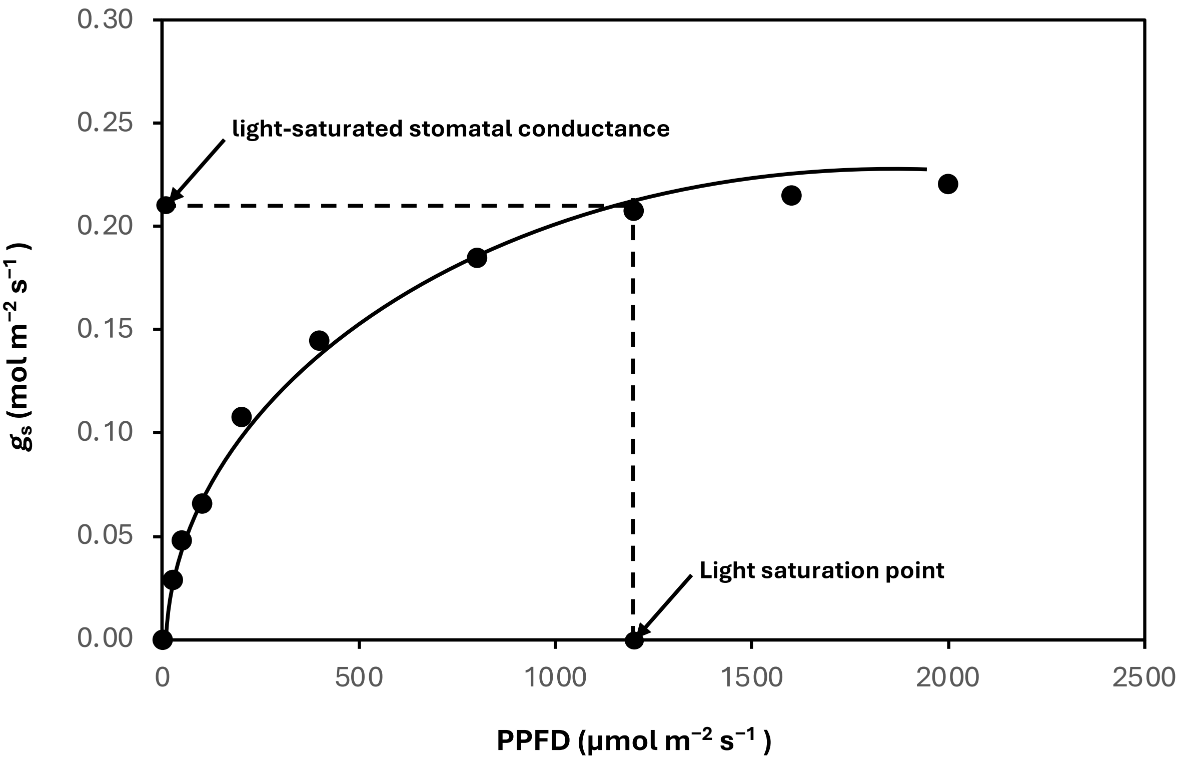

Fig. S3 Diagram of the hydraulic traits including the turgor loss point (Ψ_{TLP}), the osmotic potential at full turgor (Ψ_{FT}), the relative water content at the turgor loss point (RWC_{TLP}), and the apoplastic water fraction (AWF) at the full turgor extracted from a pressure-volume (PV) curve.

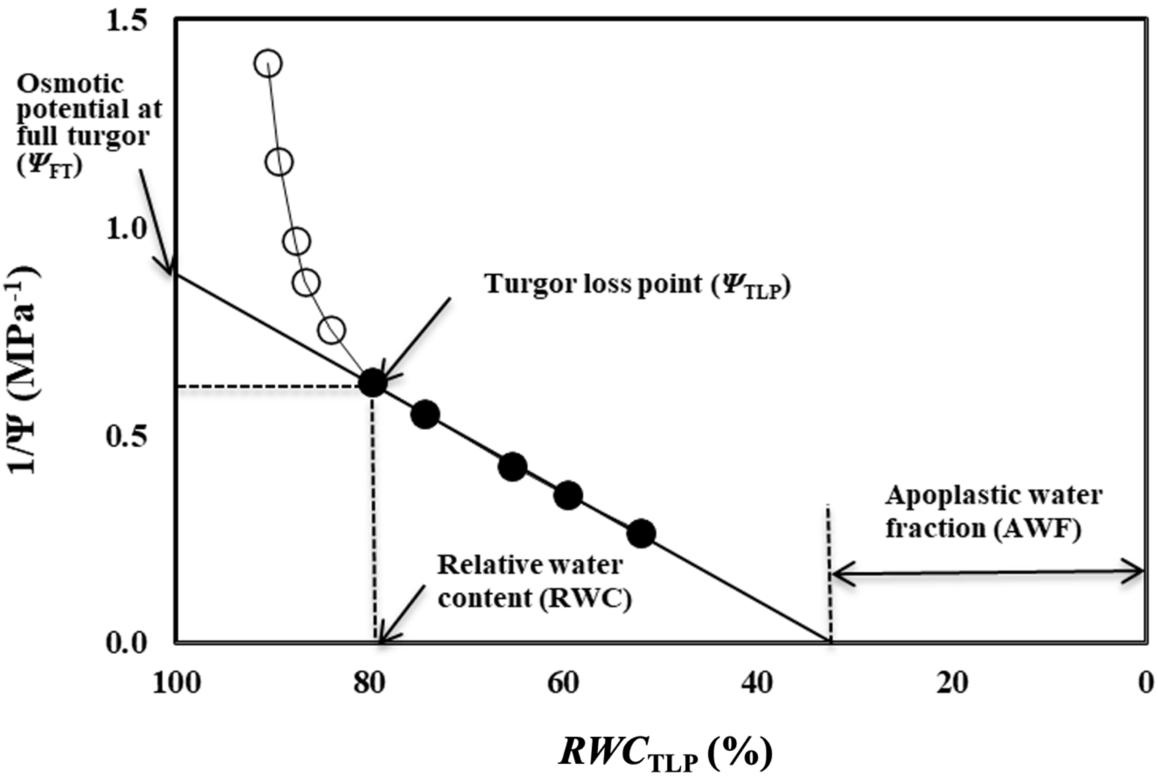

Fig. S4 Ancestral state reconstruction of bulk modulus elasticity (ε) in *A. semialata*. The measured and inferred values of bulk modulus of elasticity are mapped on a time-calibrated phylogeny of all individuals included in this study. Dot size represents the absolute values of bulk modulus of elasticity, as observed for tips and inferred for ancestral nodes. Individual names in different colours represent different photosynthetic types (C_3 in black, C_3–C_4 in grey, C_4 in white).

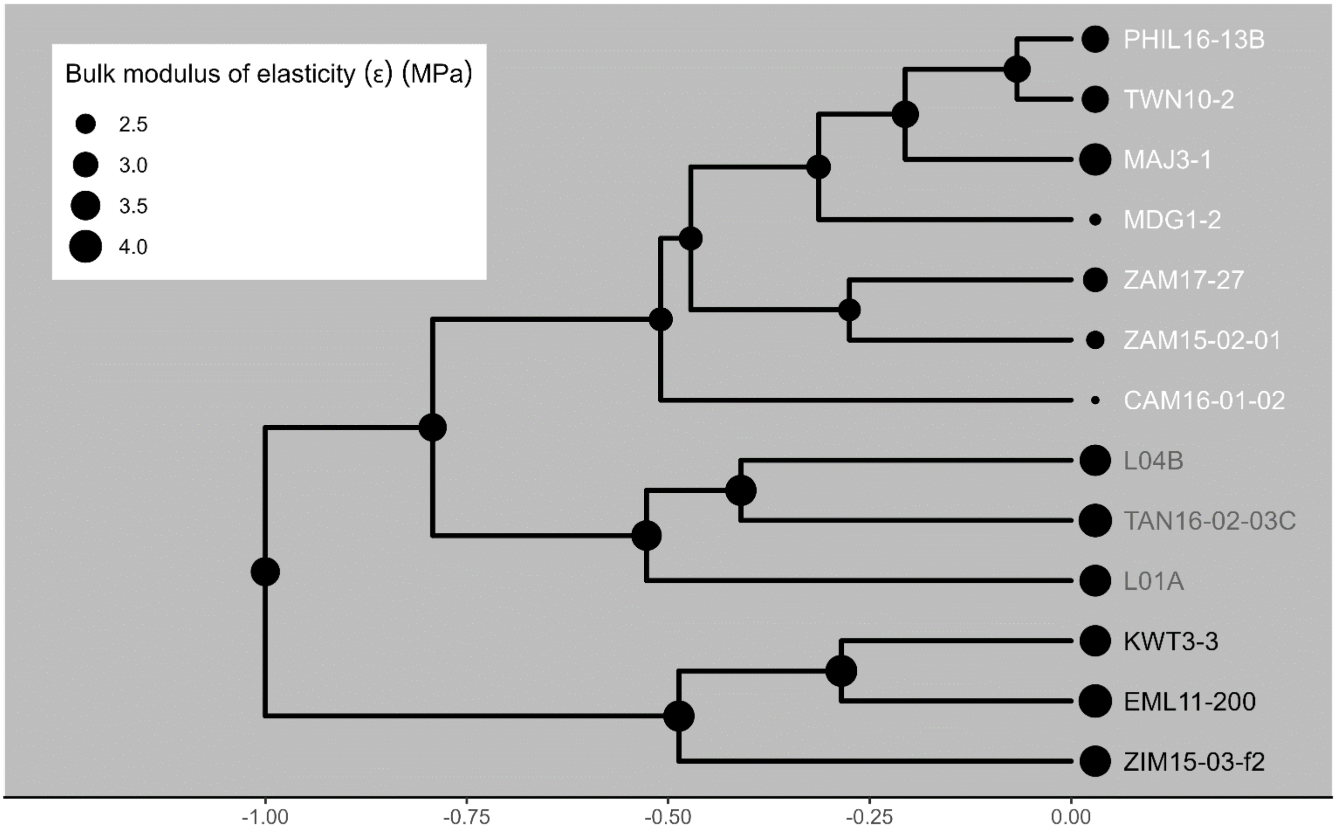

Fig. S5 Ancestral state reconstruction of leaf capacitance (C_{bulk}) in *A. semialata*. The measured and inferred values of leaf capacitance are mapped on a time-calibrated phylogeny of all individuals included in this study. Dot size represents the absolute values of leaf capacitance, as observed for tips and inferred for ancestral nodes. Individual names in different colours represent different photosynthetic types (C_3 in black, C_3–C_4 in grey, C_4 in white).

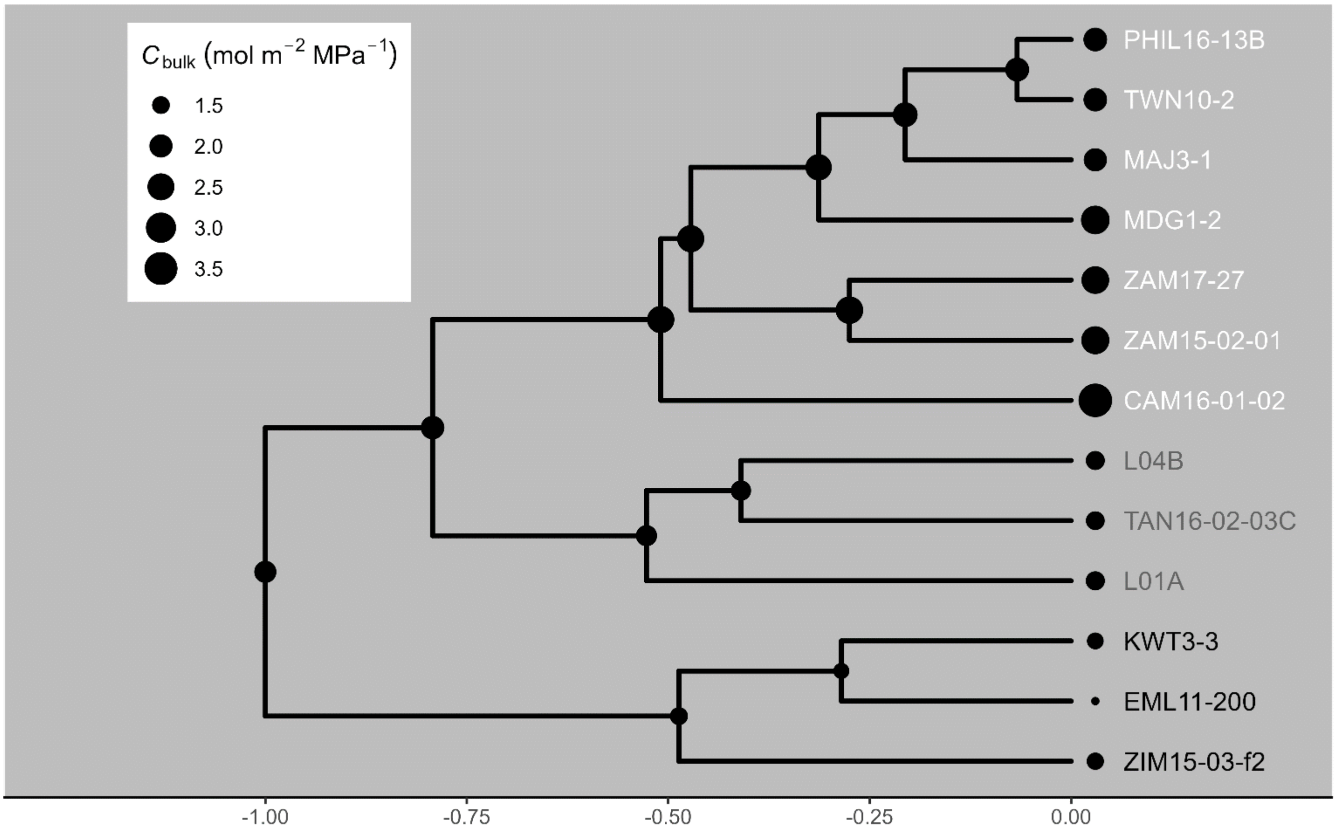

Fig. S6 Ancestral state reconstruction of turgor loss point (Ψ_{TLP}) in *A. semialata*. The measured and inferred values of the turgor loss point are mapped on a time-calibrated phylogeny of all individuals included in this study. Dot size represents the absolute values of the turgor loss point, as observed for tips and inferred for ancestral nodes. Individual names in different colours represent different photosynthetic types (C_3 in black, C_3–C_4 in grey, C_4 in white).

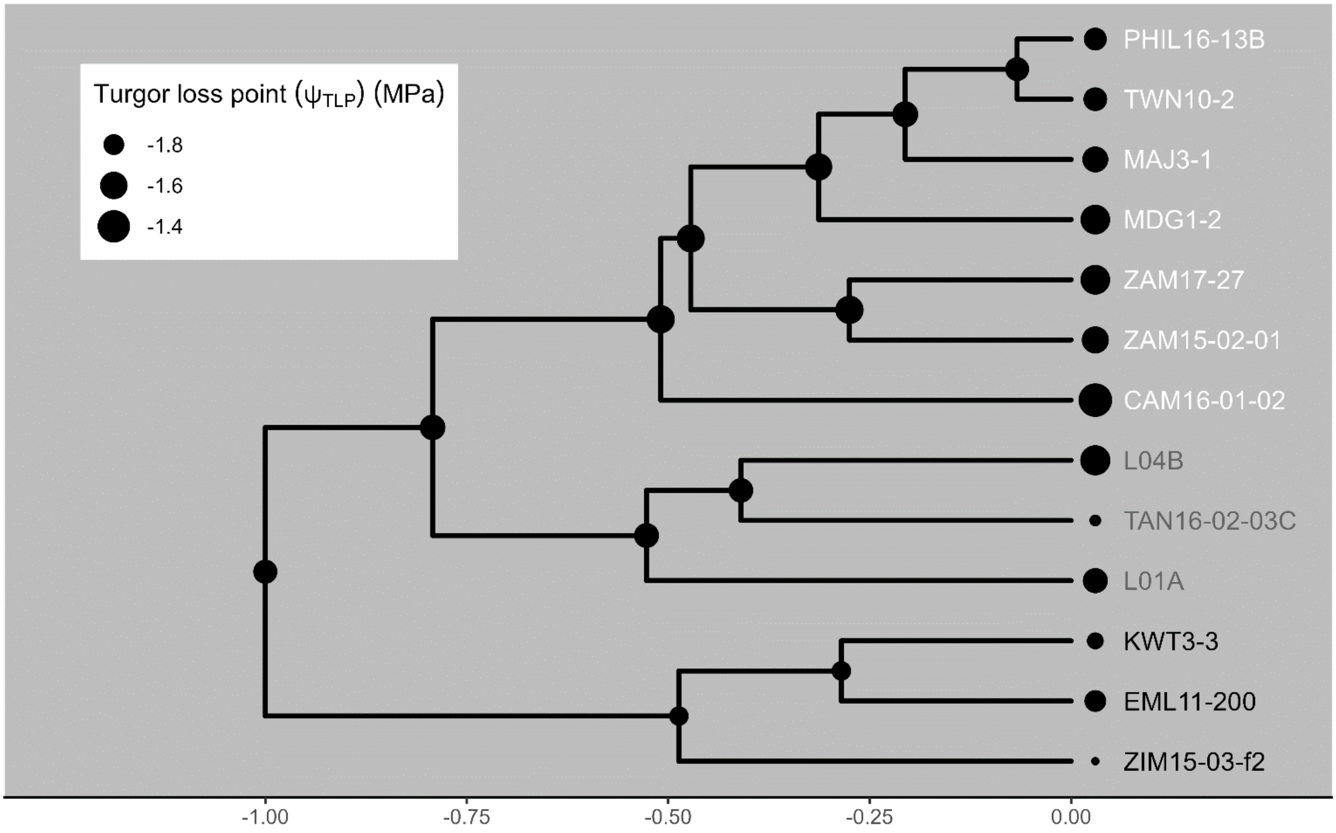

Fig. S7 Relationships between leaf anatomy and leaf hydraulics, including the total bundle sheath area to leaf width ratio (BSA per leaf width) and turgor loss point (Ψ_{TLP}) (a), bulk modulus of elasticity (ε) (b), leaf capacitance (C_{bulk}) (c), leaf water potential (Ψ_{leaf}) (d) and leaf hydraulic conductance (K_{leaf}) (e); relationships between leaf thickness (LT) and Ψ_{TLP} (f), ε (g), C_{bulk} (h), Ψ_{leaf} (i) and K_{leaf} (j); relationships between major vein density (VLA1) and Ψ_{TLP} (k), ε (l), C_{bulk} (m), Ψ_{leaf} (n) and K_{leaf} (o); and relationships between minor vein density (VLA2) and Ψ_{TLP} (p), ε (q), C_{bulk} (r), Ψ_{leaf} (s) and K_{leaf} (t) in C_3 grasses (black circles), C_3–C_4 grasses (grey circles), C_4–2x grasses (white circles), C_4–6x grasses (white squares) and C_4–12x grasses (white triangles) of *A. semialata*. Each symbol represents mean values (n = 3). Error bars indicate means ± SEs. Phylogenetic generalised least squares (PGLS) model adjusted r^2 and *p*-value results are shown.

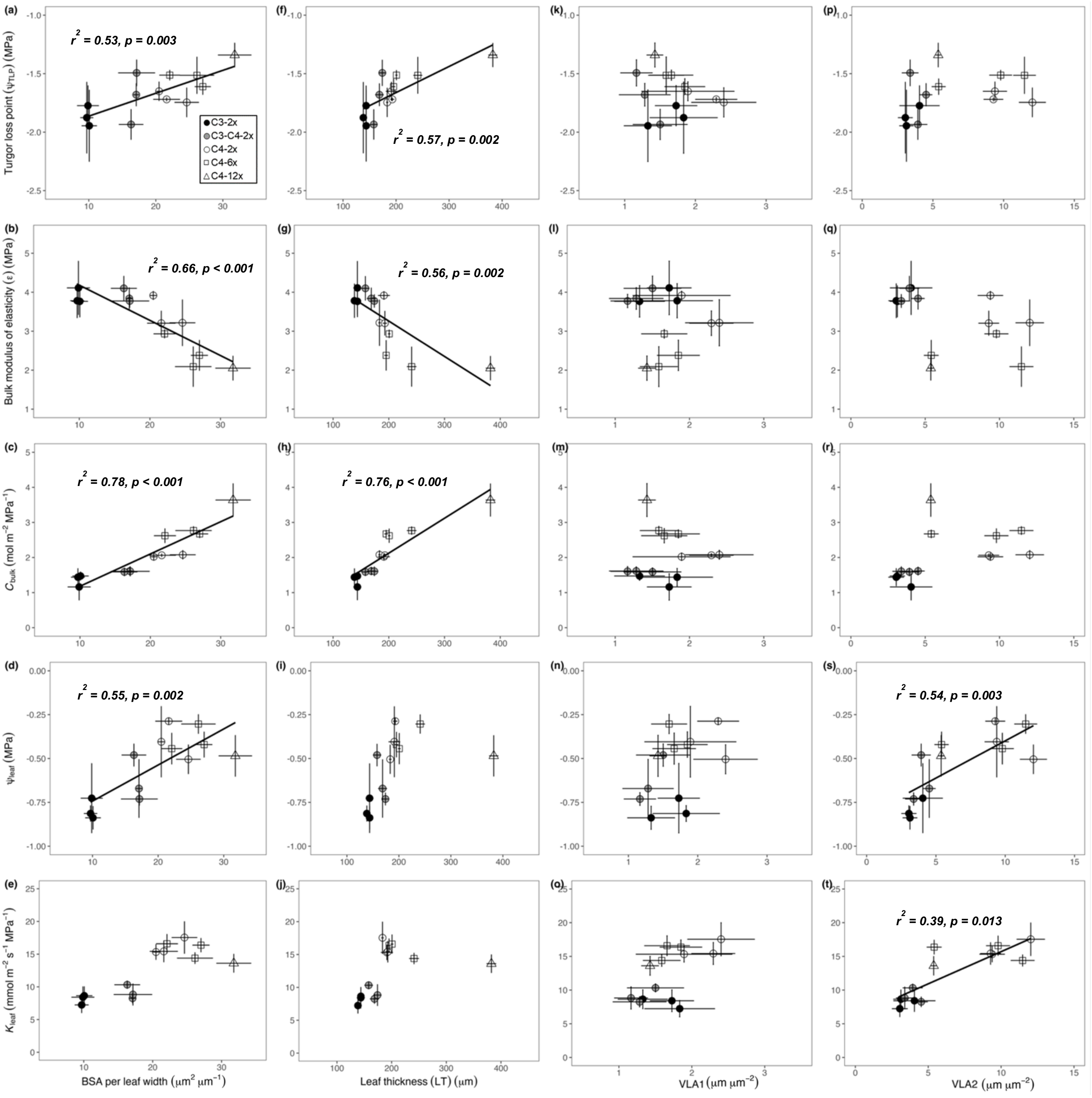

(a)
(b)
(c)
(d)
(e)
(f)
(g)
(h)
(i)
(j)
(k)
(l)
(m)
(n)
(o)
(p)
(q)
(r)
(s)
(t)
Turgor loss point (ψ_{TLP}) (MPa)
Bulk modulus of elasticity (ε) (MPa)
C_{bulk} (mol m^{-2} MPa^{-1})
ψ_{leaf} (MPa)
K_{leaf} (mmol m^{-2} s^{-1} MPa^{-1})
BSA per leaf width (μm^2 μm^{-1})
Leaf thickness (LT) (μm)
VLA1 (μm μm^{-2})
VLA2 (μm μm^{-2})
C3-2x
C3-C4-2x
C4-2x
C4-6x
C4-12x
r^2 = 0.53, p = 0.003
r^2 = 0.57, p = 0.002
r^2 = 0.66, p < 0.001
r^2 = 0.56, p = 0.002
r^2 = 0.78, p < 0.001
r^2 = 0.76, p < 0.001
r^2 = 0.55, p = 0.002
r^2 = 0.54, p = 0.003
r^2 = 0.39, p = 0.013

Fig. S8 Relationship between bundle sheath cell and leaf hydraulics, including inner bundle sheath area (IBSA) per leaf width and leaf capacitance (C_{bulk}) (a), leaf water potential (Ψ_{leaf}) (b), turgor loss point (Ψ_{TLP}) (c); the relationship between parenchymatous bundle sheath area (PBSA) per leaf width and C_{bulk} (d), Ψ_{leaf} (e), Ψ_{TLP} (f) in C_3 grasses (black circles), C_3–C_4 grasses (grey circles), C_4–2x grasses (white circles), C_4–6x grasses (white squares) and C_4–12x grasses (white triangles) of *A. semialata*. Each symbol represents mean values (n = 3). Error bars indicate means ± SEs. Phylogenetic generalised least squares (PGLS) model adjusted r^2 and p-value results are shown.

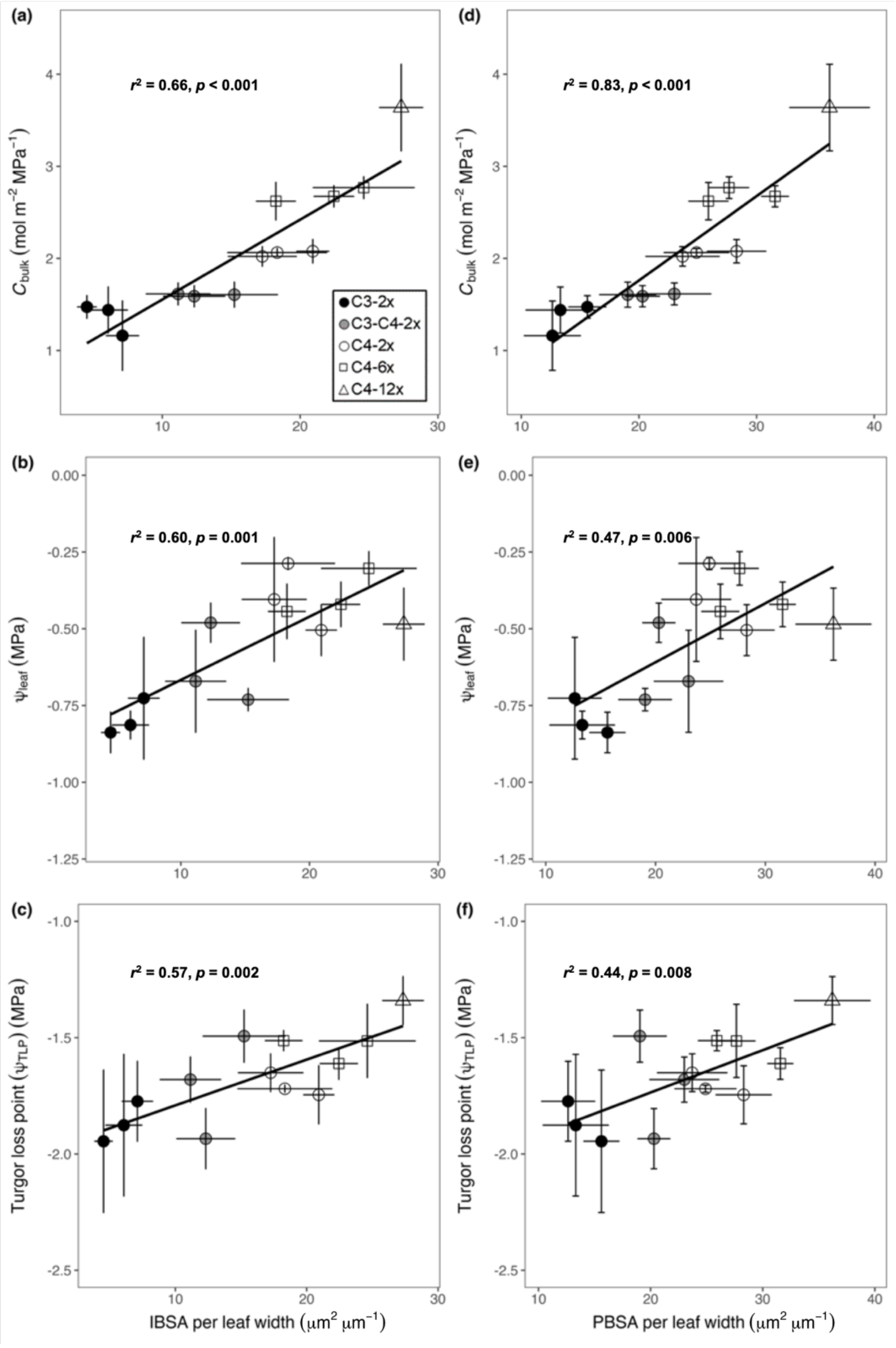

Fig. S9 Interactive effect of total bundle sheath area to leaf width ratio (BSA per leaf width) and leaf thickness on leaf capacitance (C_{bulk}) in *A. semialata*. The effect of BSA per leaf width on C_{bulk} when fixing leaf thickness (a); the effect of leaf thickness on C_{bulk} when fixing BSA per leaf width (b) in C_3 grasses (black circles), C_3–C_4 grasses (grey circles), C_4–2x grasses (white circles), C_4–6x grasses (white squares) and C_4–12x grasses (white triangles) of *A. semialata*. Each symbol represents mean values (n = 3). Error bars indicate means ± SEs. Phylogenetic generalised least squares (PGLS) model adjusted r^2 and *p*-value results are shown.

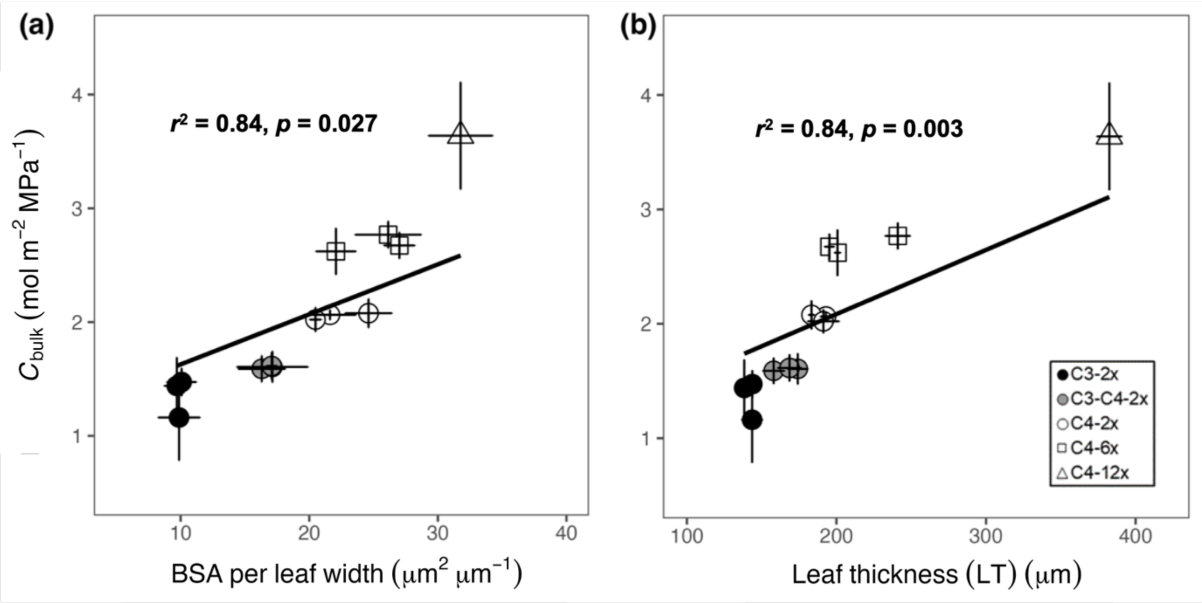

Fig. S10 Relationships between PC1 and leaf anatomy or hydraulics, including leaf thickness (a), total bundle sheath area to leaf width ratio (BSA per leaf width) (b), and minor vein density (VLA2) (c); relationships between PC1 and leaf water potential (Ψ_{leaf}) (d), leaf hydraulic conductance (K_{leaf}) (e), and leaf capacitance (C_{bulk}) (f) in C_3 grasses (black circles), C_3–C_4 grasses (grey circles), C_4–2x grasses (white circles), C_4–6x grasses (white squares) and C_4–12x grasses (white triangles) of *A. semialata*. Each symbol represents mean values (n = 3). Error bars indicate means ± SEs. Phylogenetic generalised least squares (PGLS) model adjusted r^2 and *p*-value results are shown.

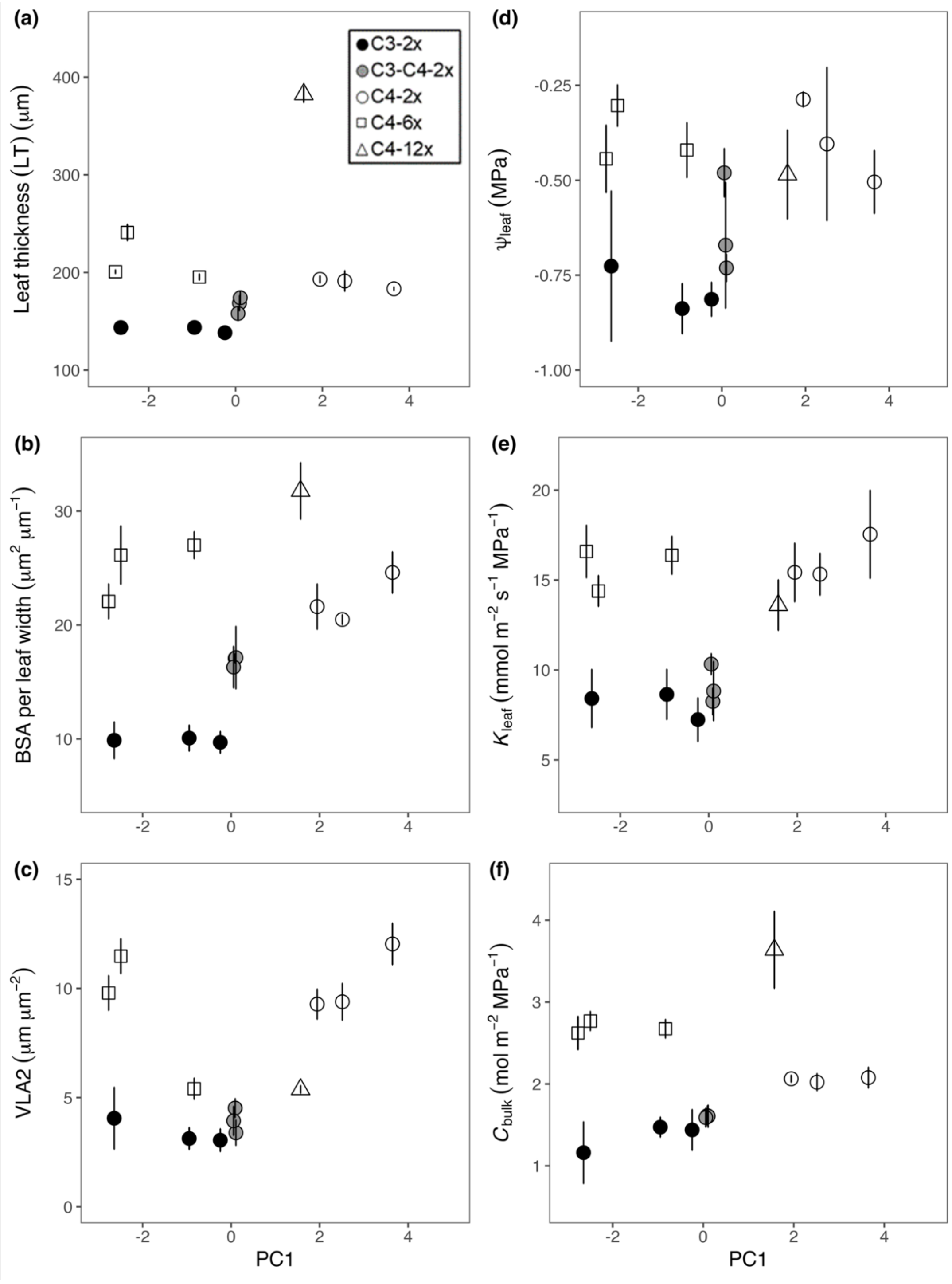

Fig. S11 Leaf cross sections representing 13 populations of *A. semialata*. (a) EML11-200, C_3 diploid plants from South Africa; (b) KWT3-3, C_3 diploid plants from South Africa; (c) ZIM15-03-f2, C_3 diploid plants from Zimbabwe; (d) L01A, C_3–C_4 diploid plants from Tanzania; (e) L04B, C_3–C_4 diploid plants from Tanzania; (f) TAN16-02-03C, C_3–C_4 diploid plants from Tanzania; (g) MAJ3-1, C_4 diploid plants from Madagascar; (h) PHIL16-13B, C_4 diploid plants from Philippines; (i) TWN10-2, C_4 diploid plants from Taiwan; (j) MDG1-2, C_4 hexaploid plants from South Africa; (k) ZAM15-02-01, C_4 hexaploid plants from Zambia; (l) ZAM17-27, C_4 hexaploid plants from Zambia; (m) CAM16-01-02, C_4 dodecaploid plants from Cameroon. Scale bar: 100 µm.

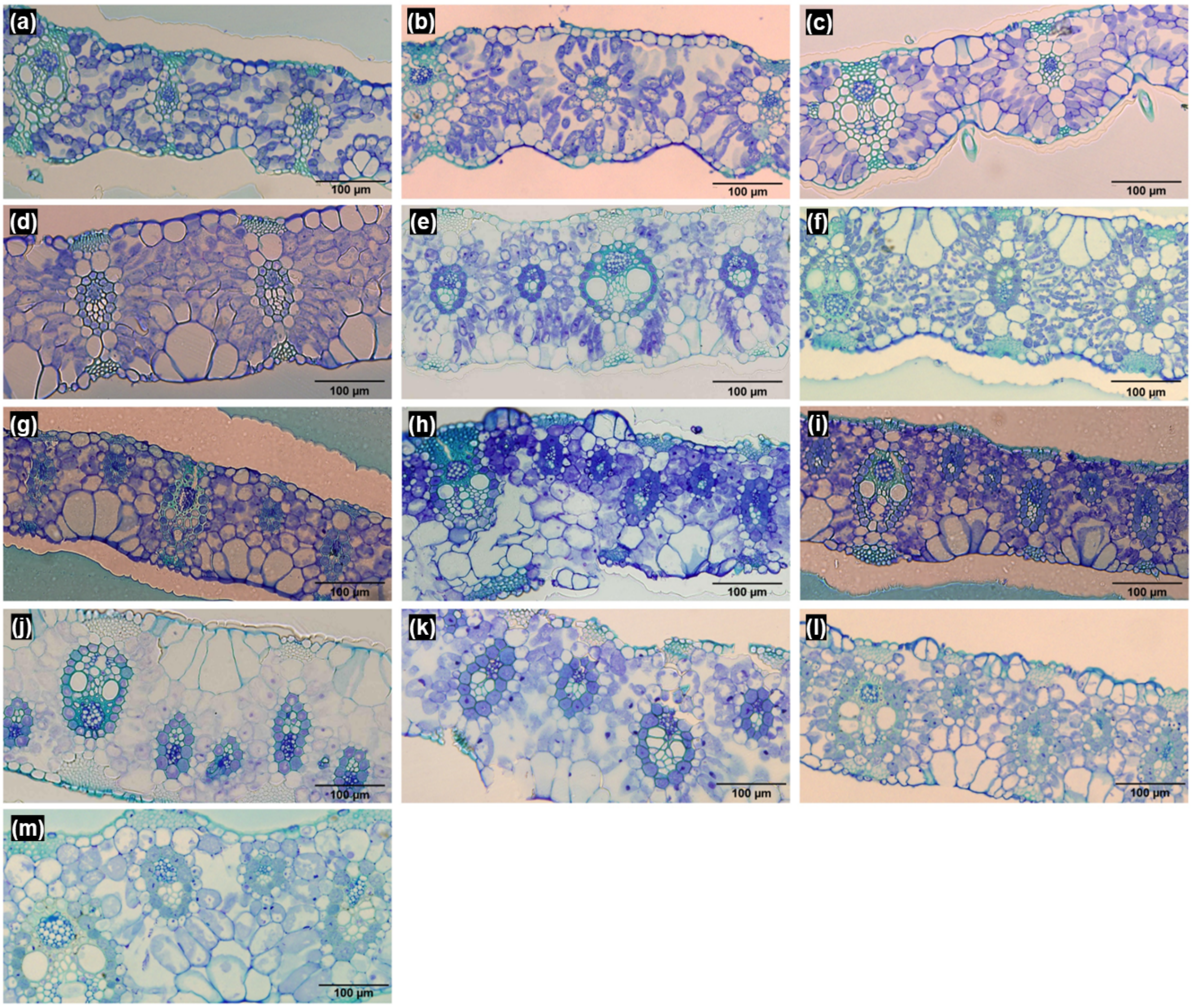

Fig. S12 Ancestral state reconstruction of leaf hydraulic conductance (K_{leaf}) in *A. semialata*. The measured and inferred values of leaf hydraulic conductance are mapped on a time-calibrated phylogeny of all individuals included in this study. Dot size represents the absolute values of leaf hydraulic conductance, as observed for tips and inferred for ancestral nodes. Individual names in different colours represent different photosynthetic types (C_3 in black, C_3–C_4 in grey, C_4 in white).

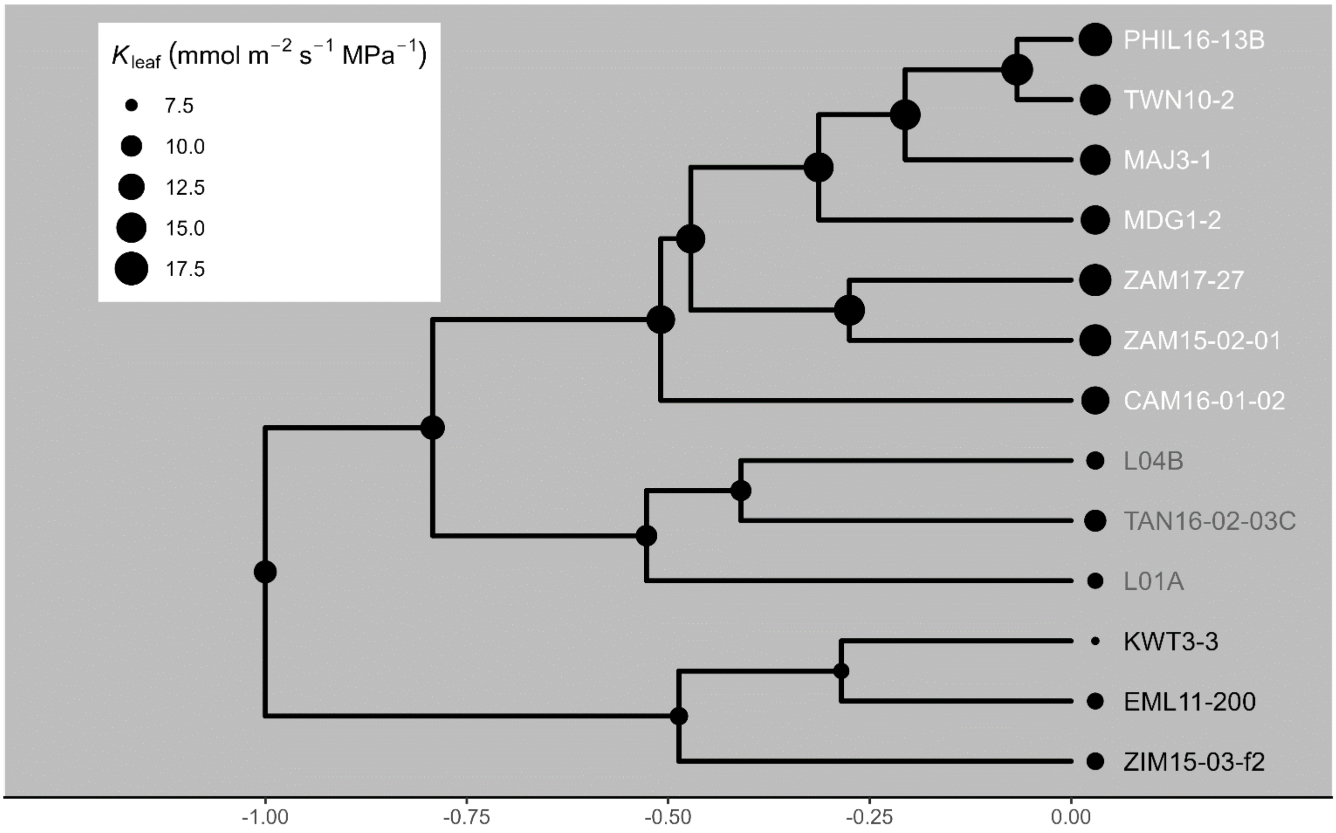

Fig. S13 Ancestral state reconstruction of leaf thickness (LT) in *A. semialata*. The measured and inferred values of LT are mapped on a time-calibrated phylogeny of all individuals included in this study. Dot size represents the absolute values of LT, as observed for tips and inferred for ancestral nodes. Individual names in different colours represent different photosynthetic types (C_3 in black, C_3–C_4 in grey, C_4 in white).

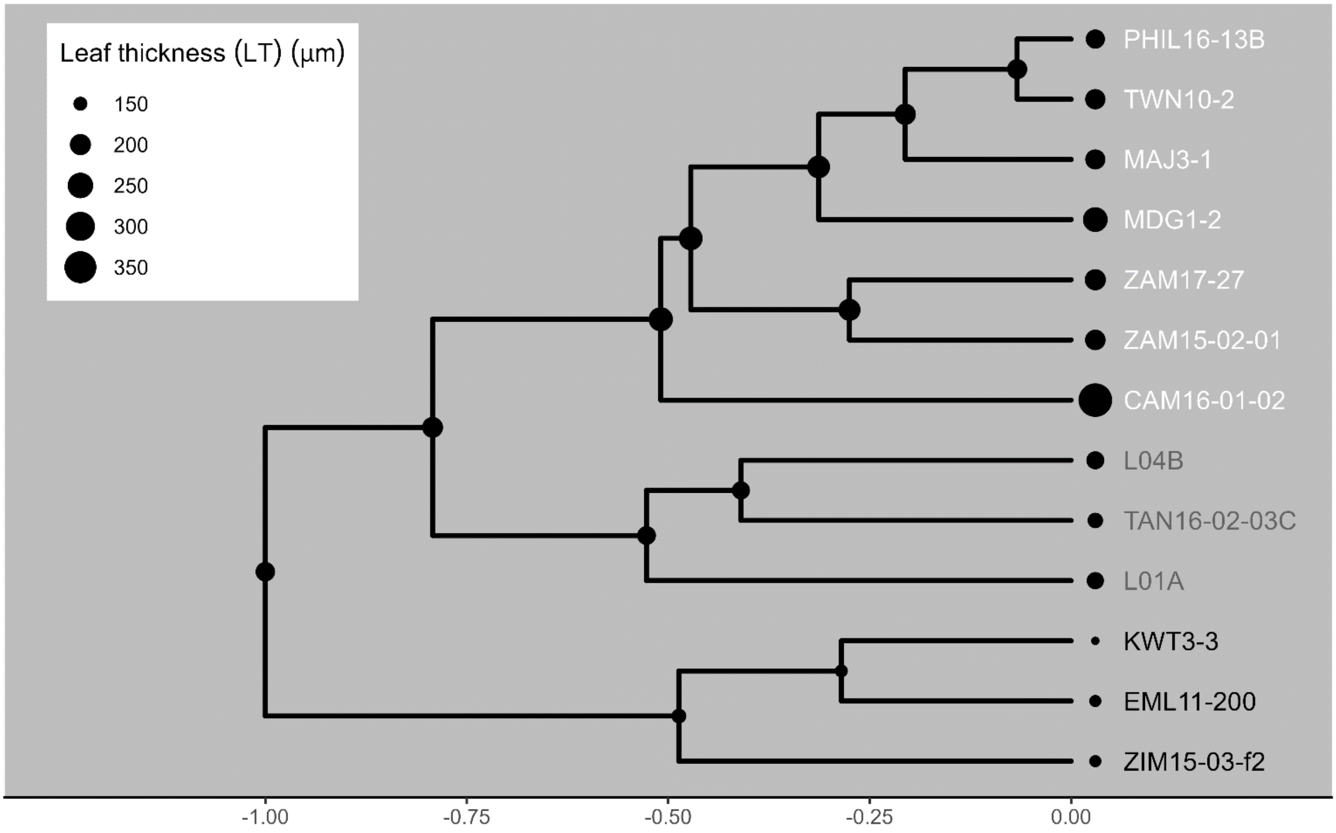

Fig. S14 Ancestral state reconstruction of the total bundle sheath area to leaf width ratio (BSA per leaf width) in *A. semialata*. The measured and inferred values of BSA per leaf width are mapped on a time-calibrated phylogeny of all individuals included in this study. The dot size represents the absolute values of BSA per leaf width, as observed for tips and inferred for ancestral nodes. Individual names in different colours represent different photosynthetic types (C_3 in black, C_3–C_4 in grey, C_4 in white).

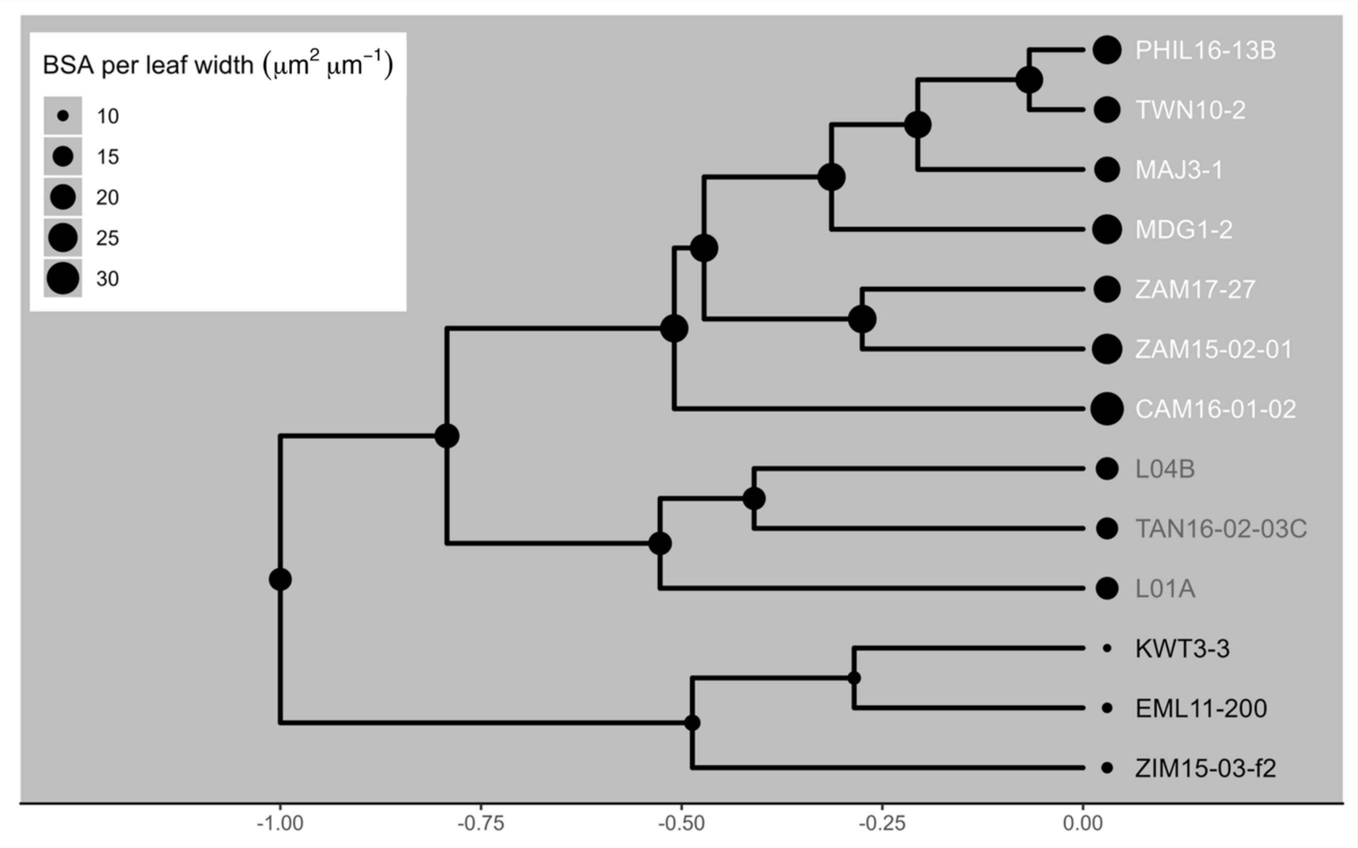

Fig. S15 Ancestral state reconstruction of minor leaf vein density (VLA2) in *A. semialata*. The measured and inferred values of VLA2 are mapped on a time-calibrated phylogeny of all individuals included in this study. Dot size represents the absolute values of VLA2, as observed for tips and inferred for ancestral nodes. Individual names in different colours represent different photosynthetic types (C_3 in black, C_3–C_4 in grey, C_4 in white).

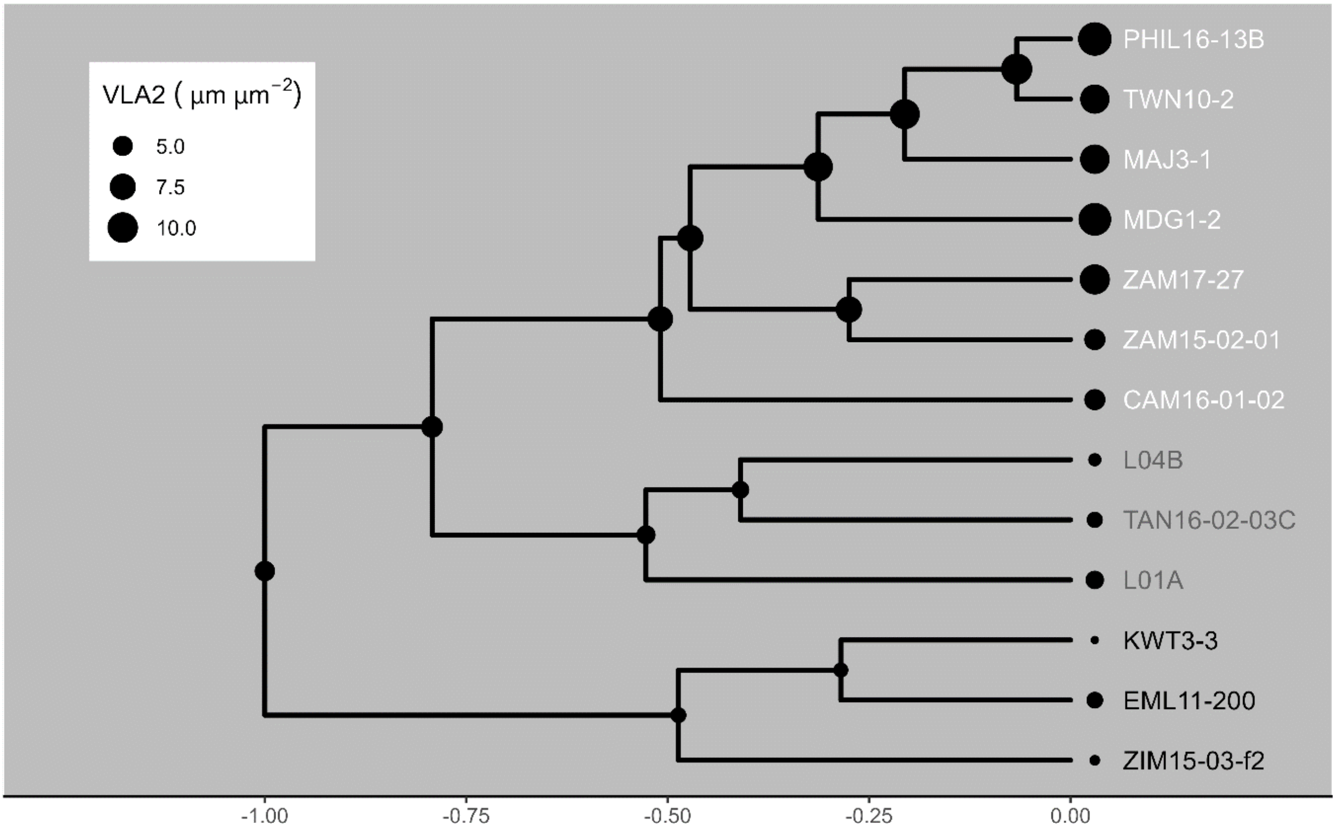

References

Bianconi ME, Dunning LT, Curran EV, Hidalgo O, Powell RF, Mian S, Leitch IJ, Lundgren MR, Manzi S, Vorontsova MS. 2020. Contrasted histories of organelle and nuclear genomes underlying physiological diversification in a grass species. *Proceedings of the Royal Society B* **287**(1938): 20201960.

Lundgren MR, Christin PA, Escobar EG, Ripley BS, Besnard G, Long CM, Hattersley PW, Ellis RP, Leegood RC, Osborne CP. 2016. Evolutionary implications of C_3–C_4 intermediates in the grass *Alloteropsis semialata*. *Plant, Cell & Environment* **39**(9): 1874-1885.

Lundgren MR, Dunning LT, Olofsson JK, Moreno-Villena JJ, Bouvier JW, Sage TL, Khoshravesh R, Sultmanis S, Stata M, Ripley BS. 2019. C_4 anatomy can evolve via a single developmental change. *Ecology Letters* **22**(2): 302-312.

Olofsson JK, Curran EV, Nyirenda F, Bianconi ME, Dunning LT, Milenkovic V, Sotelo G, Hidalgo O, Powell RF, Lundgren MR. 2021. Low dispersal and ploidy differences in a grass maintain photosynthetic diversity despite gene flow and habitat overlap. *Molecular Ecology* **30**(9): 2116-2130.
